# Cell cycle-dependent regulation of the RNA-binding protein Staufen1

**DOI:** 10.1093/nar/gku506

**Published:** 2014-06-07

**Authors:** Karine Boulay, Mehdi Ghram, Wildriss Viranaicken, Véronique Trépanier, Stéphanie Mollet, Céline Fréchina, Luc DesGroseillers

**Affiliations:** Département de Biochimie, Faculté de médecine, Université de Montréal, 2900 Edouard Montpetit, Montréal, QC, H3T 1J4, Canada

## Abstract

Staufen1 (Stau1) is a ribonucleic acid (RNA)-binding protein involved in the post-transcriptional regulation of gene expression. Recent studies indicate that Stau1-bound messenger RNAs (mRNAs) mainly code for proteins involved in transcription and cell cycle control. Consistently, we report here that Stau1 abundance fluctuates through the cell cycle in HCT116 and U2OS cells: it is high from the S phase to the onset of mitosis and rapidly decreases as cells transit through mitosis. Stau1 down-regulation is mediated by the ubiquitin-proteasome system and the E3 ubiquitin ligase anaphase promoting complex/cyclosome (APC/C). Stau1 interacts with the APC/C co-activators Cdh1 and Cdc20 via its first 88 N-terminal amino acids. The importance of controlling Stau1^55^ levels is underscored by the observation that its overexpression affects mitosis entry and impairs proliferation of transformed cells. Microarray analyses identified 275 Stau1^55^-bound mRNAs in prometaphase cells, an early mitotic step that just precedes Stau1 degradation. Interestingly, several of these mRNAs are more abundant in Stau1^55^-containing complexes in cells arrested in prometaphase than in asynchronous cells. Our results point out for the first time to the possibility that Stau1 participates in a mechanism of post-transcriptional regulation of gene expression that is linked to cell cycle progression in cancer cells.

## INTRODUCTION

It is now well accepted that post-transcriptional mechanisms of gene regulation are active to properly link protein synthesis to cell needs (1,2). It was proposed that ribonucleic acid (RNA)-binding proteins and non-coding RNAs tag and group functionally related messenger RNAs (mRNAs) into RNA regulons to ensure that proteins involved in a specific pathway are coordinately translated at the right time (1). As a consequence, even a slight modulation in the expression and/or activity of an RNA-binding protein is likely to profoundly affect the pathway(s) controlled by its bound mRNAs. In mammals, Staufen1 (Stau1) is a key factor in the post-transcriptional regulation of gene expression (3–6). Stau1 is a double-stranded RNA-binding protein that is ubiquitously expressed and alternative splicing of its mRNA generates protein isoforms of 55 kDa (Stau1^55^) and 63 kDa (Stau1^63^) (7,8). Stau1 is involved in several post-transcriptional mechanisms that control gene expression including mRNA transport (4,5,9), translation (3,10,11), decay (6,12), nuclear export (13,14) and splicing (14). All these functions are likely very important for cell physiology as compelling data indicate that Stau1 is involved in cell differentiation (12,15–20), dendritic spine morphogenesis (9,21) and long-term synaptic plasticity (21), a cellular mechanism for long term memory. Therefore, Stau1 is a multifunctional protein and many of its functions are related to post-transcriptional regulation of gene expression.

Recent studies identified Stau1-bound mRNAs and the *cis*-acting sequences responsible for Stau1 association (10,11,22–24). Essentially, two broad classes of Stau1-bound mRNAs were identified. A first class of transcripts contains inverted Alu sequences in their 3′UTR whereas a second class with GC-rich region forms high secondary structure-forming propensity in the coding region and 3′UTR. A large fraction of these mRNAs are associated with Stau1 on translating ribosomes (10,11,25). Genome-wide analyses reveal that Stau1-bound mRNAs code for proteins with heterogeneous functions mainly related to transcription, translation, cell growth and regulation of cell cycle (10,11,22–24). Therefore, modulation of Stau1 level by cell cycle effectors may dictate the post-transcriptional expression of its bound transcripts and therefore may contribute to the control of cell proliferation. Indeed, Stau1 down-regulation protects several transcripts from degradation (12) while Stau1 overexpression increases ribosome occupancy of high-GC-content transcripts (10). Moreover, experimental evidence supported the hypothesis that Stau1 target selection is strongly influenced by Stau1 levels, probably due to the low complexity and redundancy of *cis*-acting binding sequences (23).

Many cell cycle effectors that maintain a tight control on the expression of downstream molecules involved in the progression of the cycle and in cell division are timely expressed through the cell cycle (26,27). To achieve their differential expression, these proteins are often subjected to cell cycle stage-specific targeted degradation via the ubiquitin-proteasome system (UPS), a general mechanism that controls their function and largely contributes to the unidirectionality of the cell cycle (28). The anaphase promoting complex/cyclosome (APC/C) and SKP1-Cul1-Fbox (SCF) complex are two major E3 ubiquitin ligases involved in the specificity of this process. Indeed, they play central roles from anaphase to the late G_1_ phase and from the end of G_1_ phase to early mitosis, respectively (29). APC/C activity is regulated through the cell cycle by its association with either of two activator subunits, Cdc20 and Cdh1. While Cdc20 activates APC/C during the metaphase to anaphase transition, Cdh1 maintains its activation from anaphase until the end of G_1_ phase (30). Cdc20 and Cdh1 selectively recruit APC/C substrates bearing sequence recognition motifs including the destruction boxes (D-box) and the KEN-box consensus sequences (31–33).

In this paper, we show that Stau1 levels decrease during mitosis transit via APC/C- and UPS-dependent mechanisms. Time-specific Stau1 degradation is likely important since imbalance of Stau1^55^ levels impairs mitosis and cell proliferation. We also provide evidence that at least some transcripts are differentially associated with Stau1^55^ in prometaphase and asynchronous cells.

## MATERIALS AND METHODS

### Plasmids, antibodies and reagents

Plasmids coding for HA-Cdh1 and HA-Cdc20 were obtained from Dr Michele Pagano (34) and GFP-Ubiquitin from Dr Michel Bouvier (35). FLAG-YFP, FLAG-Cdh1 and FLAG-Cdc20 were generated by polymerase chain reaction (PCR) amplification of pCMV-YFP-topaz (Packard Bioscience/PerkinElmer LifeSciences), HA-Cdh1 and HA-Cdc20 and the resulting fragments were cloned in pFLAG-CMV6a (Sigma) that contains a cytomegalovirus promoter. Plasmids coding for pcDNA3-RSV-Stau1^55^-FLAG and pcDNA3-RSV-Stau1^Δ2^-FLAG (identified as pcDNA-RSV-Stau1^ΔNt88^-Flag) and driven by a Rous Sarcoma virus promoter were previously described (36). Mutation of the Stau1 D-box sequence to generate pcDNA3-RSV-Stau1^Dmut^-FLAG was done by PCR-based site-directed mutagenesis using pcDNA3-RSV-Stau1^55^-FLAG as a template. In this plasmid, the D-box consensus sequence R^375^XXL^378^ was changed for A^375^XXA^378^. To generate plasmids used for retroviral-mediated gene transfer, the coding sequences of Stau1^55^-FLAG and Stau1^55^-HA_3_ were amplified by PCR and the resulting fragments were cloned in the BglII site of the retroviral vector pMSCVpuro (Clonetech laboratories) that contains the Murine Stem Cell Virus promoter. To construct the pMSCVpuro-Stau1^55^-FLAG_3_ plasmid, oligonucleotides (5′-GGCCTTGACTACAAAGACCATGACGGTGATTATAAAGATCATGACATCGACTACAAGGATGACGATGACAAG-3′ and 5′-GGCCCTTGTCATCGTCATCCTTGTAGTCGATGTCATGATCTTTATAATCACCGTCATGGTCTTTGTAGTCAA-3′) were hybridized and then inserted into the NotI sites of pMSCVpuro-Stau1^55^-HA_3_ in replacement of the HA_3_-tag. pMSCVpuro-Stau1^Dmut^-FLAG_3_ was constructed as described above using pMSCVpuro-Stau1^55^-FLAG_3_ as a template. To construct pMSCVpuro-Stau1^Δ2^-FLAG_3_, the coding sequence of Stau1^Δ2^-FLAG was first amplified by PCR and cloned in pMSCVpuro. Then, the EcoRI fragment of pMSCVpuro-Stau1^55^-FLAG_3_ (containing the C-terminal coding sequence of Stau1^55^ fused to the FLAG_3_ tag) was cloned into EcoRI-digested pMSCVpuro-Stau1^Δ2^-FLAG plasmid. Note that pMSCVpuro-Stau1^55^-FLAG_3_, pMSCVpuro-Stau1^Dmut^-FLAG_3_ and pMSCVpuro-Stau1^Δ2^-FLAG_3_ were also used for transient transfections.

Antibodies against Cyclin A (CY-A1), β-Actin (Ac-74), FLAG (M2), HA (rabbit polyclonal) were purchased from Sigma; against phospho-Histone H3 (Ser10) (D2C8) and ribosomal protein S6 from Cell Signaling; against MPM2 and Aurora A from Abcam; against GFP from Roche Applied Science; anti-cyclin B1 from Santa Cruz and anti-PARP1 from New England Biolabs. Monoclonal antibodies against Stau1 (3) and HA (8) were previously described. SmartPool On-TARGETplus-Non-targeting siRNA #1 (control) and On-TARGETplus SMARTpool-Human Cdc20 siRNAs were purchased from ThermoFisher/Dharmacon.

### Cell culture and synchronization

The human cell lines hTert-RPE1, IMR90, HEK293T, U2OS and phoenix retroviral packaging cells were cultured in Dulbecco modified Eagle's medium (Invitrogen) supplemented with 10% cosmic calf serum (HyClone) or fetal bovine serum (Wisent), 100 μg/ml streptomycin and 100 units/ml penicillin (Wisent) (hereafter referred to as complete DMEM). HCT116 cells were maintained in McCoy's medium (Invitrogen) supplemented with 10% fetal bovine serum, 100 μg/ml streptomycin and 100 units/ml penicillin. Cells were cultured at 37°C under a 5% CO_2_ atmosphere. When required, 10 μM MG132 (Enzo Life Sciences) was added to the medium for the indicated periods before harvesting the cells for western blots.

HCT116 and U2OS cells were synchronized at the G_1_/S border using a thymidine double-block protocol (37). Briefly, cells were treated with 2 mM thymidine for 16 h and released for 8 h in fresh medium before the second block was performed for another 16 h with 2 mM thymidine. Cells were then washed three times in phosphate buffer saline (PBS) and either collected (hours after treatment = 0) or released in fresh medium for different time periods. For synchronization in prometaphase, HCT116 and U2OS cells were synchronized as described above. After that, cells were released in fresh thymidine free medium for 3 h then treated with 50 ng/ml nocodazole for 8 h. Round mitotic cells were recovered by gentle shaking of the culture dish (shake-off) (hours after release = 0) and re-plated in fresh medium for different time periods. HCT116 cells were also synchronized at the G_2_/M phase border using 10 μM of the Cdk1 inhibitor RO-3306 (38) (Enzo Life Sciences Inc.) for 18 h. Cells were then washed three times in PBS and either collected (hours after treatment = 0) or released in fresh medium for different time periods. For synchronization in prometaphase, HEK293T cells were treated with 50 ng/ml nocodazole for 18 h.

### DNA transfection and retroviral gene transfer

For transient expression, cells were transfected with lipofectamine 2000 according to the manufacturer's instructions (Invitrogen). Alternatively, cells were infected with retroviral particles as described previously (39) and selected with 3 μg/ml puromycin for 2 days.

### Flow cytometry analysis

Cell cycle distribution was determined by fluorescence activated cell sorting (FACS). Cells were trypsinized and fixed in 70% ethanol at −20°C for at least 2 h. Cells were resuspended in PBS containing 40 μg/ml propidium iodide and 100 μg/ml RNase A. After incubation for 1 h at 37°C cells were characterized. Data was acquired using a BD LSRII apparatus and analyzed using the FlowJo software. For each experiment, 10^4^ cells were analyzed.

### Growth curves and colony formation assays

Cells were selected with puromycin as described above. After selection, cells were plated at the same density (day = 0). For growth curve assays, cells were harvested every day and the number of cells was counted with a hemacytometer. For colony formation assays, cells were grown for a time period ranging from 10 to 14 days as indicated. Cells were then washed two times with PBS and colored with 0.5% crystal violet in 50% methanol for 10 min. After extensive washes in water, plates were dried and scanned. Colony formation was determined by measuring absorbance at 590 nm using a spectrophotometer after dissolving colonies from dried plates in a solution containing 0.1 M sodium citrate (pH 4.2) and 20% methanol.

### Apoptosis, senescence and quiescence

In parallel with the growth curve assays, infected cells were collected at day 4 after plating and tested for the presence of apoptotic, senescent and/or quiescent cells. The presence of apoptotic cells was evaluated by western blotting using the anti-PARP1 antibody whereas quiescent cells were detected by FACS after labeling of the cells with pyronin Y (Sigma) and Hoechst (Fisher) as described (40). Senescent cells were also quantified by microscopy using SA-β-galactosidase activity as described (39).

### Western blot analysis and immunoprecipitation

Total-cell extracts were prepared in lysis buffer (50 mM Tris-Cl pH 7.4, 150 mM NaCl, 2 mM MgCl_2_, 1% Triton X-100, 2 mM sodium fluoride, complete EDTA-free protease inhibitor cocktail [Roche Applied Science]), and protein concentrations were determined by Bradford assays. Cell extracts (10–20 μg) were analyzed by western blotting. Data were collected either on X-ray films (Fujifilm) or with the ChemiDoc MP Imaging System (Bio-Rad Laboratories) and the western blot signals were quantified with the ImageLab (Bio-Rad Laboratories) software.

For immunoprecipitation of FLAG-tagged proteins, transfected HEK293T cells were washed three times in PBS and protein extracts were prepared in lysis buffer 24 h post-transfection. Lysates were cleared by centrifugation at 15 000 *g* for 15 min. Immunoprecipitation of FLAG-tagged proteins was performed with anti-FLAG M2 affinity gel (Sigma-Aldrich) and the immune complexes were eluted with the FLAG peptide (Sigma-Aldrich) as previously described (41). For the analysis of Stau1^55^-HA_3_ ubiquitination by immunoprecipitation, transfected cells were lysed as described above, except that cells were treated with 20 μM MG132 for 8 h and 10 mM *N*-ethylmaleimide was added to the lysis buffer. Cleared lysates were incubated with mouse monoclonal anti-HA antibody (12CA5) for 2 h at 4°C and then with protein A-sepharose beads for an additional 2 h at 4°C. Immune complexes were washed three times with the lysis buffer and eluted from the resin by heating at 95°C for 5 min in protein loading buffer. Protein expression before immunoprecipitation and immunoprecipitated complexes-associated proteins were analyzed by sodium dodecyl sulphate-polyacrylamide gel electrophoresis (SDS-PAGE) and western blotting.

For microarray analysis and its validation, asynchronous and nocodazole-treated HEK293T cells were lysed in 50 mM Tris-Cl pH 7.5, 15 mM EGTA pH 8, 0.5% Triton X-100, 100 mM NaCl, complete EDTA-free protease inhibitor cocktail (Roche Applied Science), RNAse inhibitor (Applied Biosystems). Stau1^55^-FLAG was immunoprecipitated with anti-FLAG M2 affinity gel (Sigma-Aldrich) and the immune complexes were eluted with the FLAG peptide (Sigma-Aldrich). Immunoprecipitation of endogenous Stau1-containing complexes was performed using the monoclonal anti-Stau1 antibody or the anti-HA antibody as a control, previously crosslinked to the protein A-sepharose beads with DMP (dimethyl pimelimidate) (Thermo Scientific). Immune complexes were eluted with 100 μl of 2× sodium dodecylsulphate loading buffer. Protein expression before immunoprecipitation and immunoprecipitated complexes-associated proteins were analyzed by SDS-PAGE and western blotting.

### RNA isolation and RT-qPCR

To determine the steady state level of endogenous Stau1 expression in synchronized cells, RNA was isolated from cell extracts using the TRIZOL Reagent (Invitrogen) according to the manufacturer's procedure. RNA was resuspended in 50 μl water and digested with DNase using the TURBO deoxyribonucleic acid (DNA)-free kit (Ambion). Reverse transcription reactions were done with 750 ng of RNA, the MuLV RT enzyme and oligo-d(T) from the GeneAmp RNA PCR kit (Applied Biosystems), according to the manufacturer's procedure. Resulting complementary DNAs (cDNAs) were qPCR amplified using the LightCycler 480 SYBR Green I Master kit (Roche) and the LightCycler 480 instrument (Roche). Sense and antisense sequences of the primer pairs used for the qPCR amplification were: Stau1, 5′-TTTGTGACCAAGGTTTCGGTTGGG-3′ and 5′-TGGGCTTGTCTGTGGCTTGACTAT-3′; GAPDH, 5′-CATGTTCGTCATGGGTGTGAACCA-3′ and 5′-AGTGATGGCATGGACTGTGGTCAT-3′.

For the validation of the microarray data, RNA was isolated using the TRIZOL Reagent as above. RNA was resuspended in 50 μl (inputs) or 20 μl (IPs) of water and digested with DNase using the TURBO DNA-free kit (Ambion). The reverse transcription reactions were done with 750 ng of input RNA or 3 μl of IP RNA using the MuLV RT enzyme from the GeneAmp RNA PCR kit (Applied Biosystems), according to the manufacturer's procedure. Specific antisense primers were used to produce corresponding cDNAs: RPL22: 5′-TTGCTGTTAGCAACTACGCGCAAC-3′, APAF1: 5′-TTTGCGAAGCATCAGAATGCGGAG-3′, RB1: 5′-TGAGCACACGGTCGCTGTTACATA-3′, SPBC24: 5′-ACTCCAGAGGTAGTCGCTGATGAA-3′, JUB: 5′-AACAAGCTCCACACCCACAGAGAT-3′, MOBKL2B: 5′-TGATGTGGAGAGCTTTGGGAAGGT-3′, VHL: 5′-TGACAAACCCTGACTGAAGGCTCA-3′. Resulting cDNAs were amplified using the LightCycler 480 SYBR Green I Master kit (Roche Applied Science) and the LightCycler 480 instrument (Roche Applied Science). Antisense primers were those used in the RT reactions and sense primers were: RPL22: 5′-TGGTGACCATCGAAAGGAGCAAGA-3′, APAF1: 5′-GCAGAATCTTTGCACACGGTTGGA-3′, RB1: 5′-TCAGAAGGTCTGCCAACACCAACA-3′, SPBC24: 5′-TTATGAGTGTGAGCCAGGGATGGT-3′, JUB: 5′-GGTTGCTGCCTGTATTCCCTGTTT-3′, MOBKL2B: 5′-TACCAGTTGTGCTTCAGCCTCCTT-3′, VHL: 5′-TACATCCGTAGCGGTTGGTGACTT-3′.

### Microarray analyses

RNA was isolated from the inputs and the IPs using the TRIZOL Reagent (Invitrogen) as above. RNA was resuspended in 50 μl (inputs) or 20 μl (IPs) of water and digested with DNase using the TURBO DNA-free kit (Ambion). Biotinylated chromosomal RNA probes were synthesized by the TotalPrep RNA labeling kit (Ambion). Illumina human genome arrays (Ref- HumanWG-6_V3 Expression BeadChip comprising 48 803 probe sets) were used for hybridization according to Illumina guidelines (*n* = 3). Hybridized chips were scanned using an Illumina iScan System. Results were recorded using the BeadStudio software platform. To identify mRNAs that specifically copurify with Stau1, signal intensities obtained for specific IPs were compared with those of control IPs using the FlexArray 1.6.2 software (Blazejczyk, M., Miron, M., Nadon, R. (2007), Genome Quebec, Montreal, Canada, http://genomequebec.mcgill.ca/FlexArray). Background was corrected using negative controls. Variance stabilization (log base2), Sd correction in variance stabilizing transformation method and robust spline normalization were applied. Each probe set presenting a fold enrichment over control of more than 2.5 (*t*-test *P*-value < 0.05) was scored as a potential Stau1-associated mRNA. Complete microarray analysis results can be found in the Supplemental Data. Microarray data have been deposited in the GEO database and are available through the series accession numbers GSE51182 and GSE51183.

## RESULTS

### The levels of Stau1 protein vary during the cell cycle

**Figure 1.**
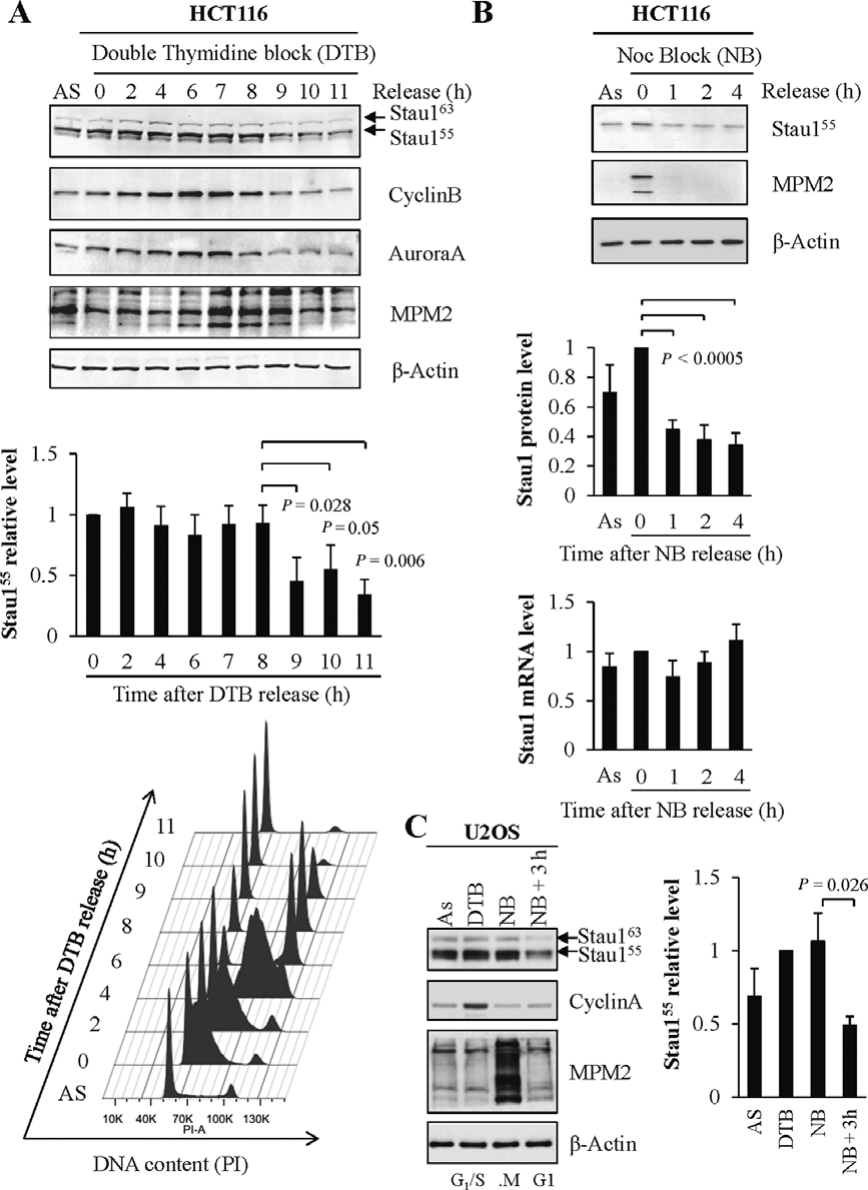
Stau1 protein levels vary during the cell cycle. (**A**) HCT116 cells were grown asynchronously (AS) or were synchronized at the G_1_/S transition by a double thymidine block (DTB) and then released by addition of fresh medium. Cell extracts were prepared at different time points post-release as indicated and analyzed by western blotting (top and middle panels). CyclinB, marker of S, G_2_ and M phases; AuroraA, marker of G_2_ and M phases; MPM2, mitosis protein monoclonal 2 detects a variety of phosphorylated proteins during mitosis. DNA content was assessed by flow cytometry analysis at the indicated time points after release from thymidine block (bottom panel). (**B**) HCT116 cells blocked in prometaphase with nocodazole (NB) were collected by shake-off and replated in fresh medium. Cell extracts were prepared at different time points and analyzed by western blotting. (Bottom) RNA was isolated from the cell extracts used for western blot analysis and the levels of mRNA were quantified by RT-qPCR. The ratios between Stau1 and GAPDH mRNA levels were calculated at each time point and the means were plotted relative to that obtained at time 0, which was arbitrarily set to 1. (**C**) U2OS cells were grown asynchronously (As) or were synchronized at the G_1_/S transition (DTB), in prometaphase (NB) or in G_1_ by a nocodazole block followed by a release of 3 h in fresh medium (NB + 3 h). Stau1 levels were monitored by western blotting. Cyclin A, marker of the G_1_/S transition. Western blot results (A, B and C) are representatives of three independently performed experiments that showed similar profiles. Statistical analyses: Quantification of the relative amounts of Stau1 protein and/or mRNA at each time point, expressed as the mean of three independent experiments. Standard deviations are shown and statistical analyses (Student's *t*-test) are indicated when significant. For protein analysis, the ratios between Stau1 and β-Actin levels were calculated at each time point and the means were plotted relative to that obtained at time 0 (A and B) or DTB (C), which was arbitrarily set to 1. Quantifications of cyclin B1, Aurora A and MPM2 are provided in Supplementary Figure S1.

To test the hypothesis that Stau1 expression is regulated in a cell cycle-dependent manner, the levels of Stau1 protein were studied in different phases of the cell cycle using synchronized cells. The colorectal carcinoma HCT116 cell line was arrested at the G_1_/S transition by a double thymidine block (DTB) and then the cells were released to allow their progression through the cell cycle. Cells were harvested at different time points after release as indicated (Figure 1A). Analysis of Stau1 levels revealed a high and stable expression during the S and G_2_ phases followed by a striking decrease ∼9 h post-DTB release, a decrease that parallels those of the mitotic markers cyclin B1 and Aurora A (Supplementary Figure S1). FACS analyses indicated that at this time point, a large proportion of the cells have reached the G_1_ phase suggesting that Stau1 down-regulation occurred during mitosis and/or when cells reached the G_1_ phase of the cell cycle (Figure 1A). Note that endogenous Stau1^55^ and Stau1^63^ behaved similarly in this experiment and in those described below. Therefore, quantification of Stau1 levels will be restricted to that of Stau1^55^ which is the major isoform.

To more precisely determine the timing of Stau1 down-regulation, we synchronized HCT116 cells in prometaphase with nodocazole. Cells were then released from the block for different time periods and Stau1 levels were quantified. Stau1 level is high in prometaphase and rapidly decreased as soon as 1 h post-release (Figure 1B) suggesting that Stau1 is down-regulated as cells progress through mitosis. HCT116 cell extracts were further analyzed by quantitative RT-PCR (RT-qPCR). While the amounts of Stau1 protein significantly decreased upon release from the nocodazole-induced arrest, the steady-state level of Stau1 mRNA was not significantly different indicating that Stau1 down-regulation during mitosis is a consequence of reduced translation and/or increased degradation of the protein. Fluctuation of Stau1 levels through the cell cycle was confirmed in the human U2OS osteosarcoma cell line (Figure 1C).

### Stau1 is degraded by the ubiquitin-proteasome system (UPS)

**Figure 2.**
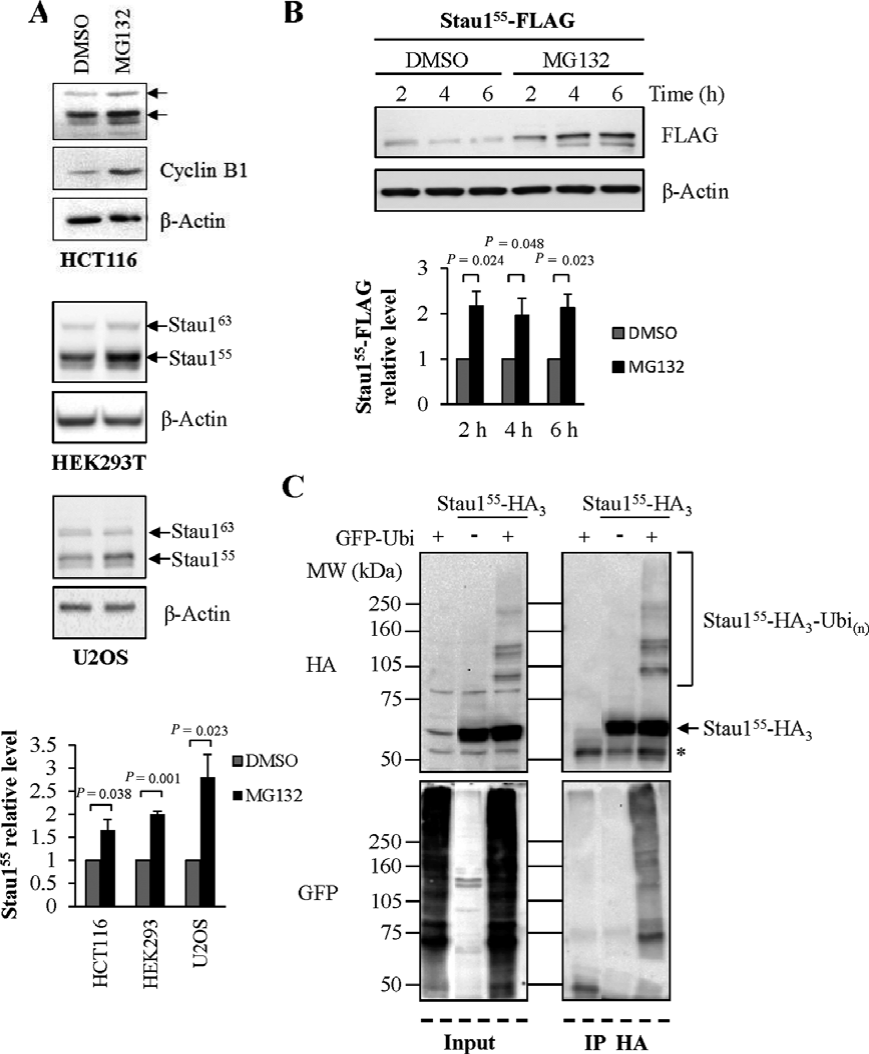
Stau1 is a substrate of the ubiquitin proteasome system. Untransfected HCT116, HEK293T and U2OS cells **(A)** and Stau1^55^-FLAG-transfected HEK293T cells **(B)** were treated for 6 h with the proteasome inhibitor MG132 (10 μM) or by DMSO (the MG132 vehicle) as control. Cell extracts were analyzed by western blotting. The ratios between Stau1 and β-Actin levels and the statistical analyses were calculated as described in the legend of Figure 1, the ratio observed in cells treated with Dimethylsulfoxide (DMSO) being arbitrarily set to 1. **(C)** HEK293T cells were transfected with plasmids coding for Stau1^55^-HA_3_ and/or GFP-Ubiquitin. Left: input. Right: Stau1^55^-HA_3_ was immunoprecipitated with a mouse monoclonal anti-HA antibody (12CA5) and co-immunoprecipitated proteins were analyzed by western blotting. *, unspecific signal. Each panel is representative of three independently performed experiments that generated similar results.

As the mitotic destruction of many proteins such as Cyclin B1 is dependent on the UPS (28), asynchronous HCT116 cells were treated with the proteasome inhibitor MG132 to determine if Stau1 is degraded by this pathway. In the presence of the drug, the amount of endogenous Stau1 augmented about 2-fold suggesting that the pharmacological treatment protected it from proteolysis (Figure 2A). As expected, the levels of Cyclin B1 also increased upon proteasome inhibition (1.99 ± 0.09; *P*-value 0.002 Student's *t*-test). Similar increases in Stau1 levels were obtained in HEK293T and U2OS cells (Figure 2A). To support this result, expression of Stau1^55^-FLAG was analyzed in HEK293T cells grown in MG132 for different time periods (Figure 2B). The protein was quickly stabilized as soon as 2 h post-MG132 treatment. These observations strongly suggest that Stau1 is degraded by the UPS.

Proteins that are destroyed by the proteasome are usually tagged by the covalent addition of poly-ubiquitin chains (28). To test if Stau1 can be modified by ubiquitin, HEK293T cells were co-transfected with plasmids coding for Stau1^55^-HA_3_ and GFP-Ubiquitin. In the total cell extracts, several slow migrating bands were detected with the anti-HA antibody, in addition to Stau1^55^-HA_3_ (Figure 2C, left). These bands were only visible when Stau1^55^-HA_3_ was co-transfected with GFP-Ubi, suggesting that the bands with reduced mobility correspond to ubiquitinated Stau1. To more precisely address this point, we immunoprecipitated Stau1^55^-HA_3_ and determined if the slow migrating Stau1^55^-HA_3_-containing bands also stained with an anti-GFP antibody (Figure 2C, right). GFP-Ubiquitin was specifically found in the Stau1^55^-HA_3_ immunoprecipitated extract and migrated as a smear, most likely because other ubiquitinated proteins were present in the immune complex. Altogether our data indicate that Stau1 is a substrate of the UPS.

### The control of Stau1 levels during mitosis is mediated by Cdh1 and Cdc20

**Figure 3.**
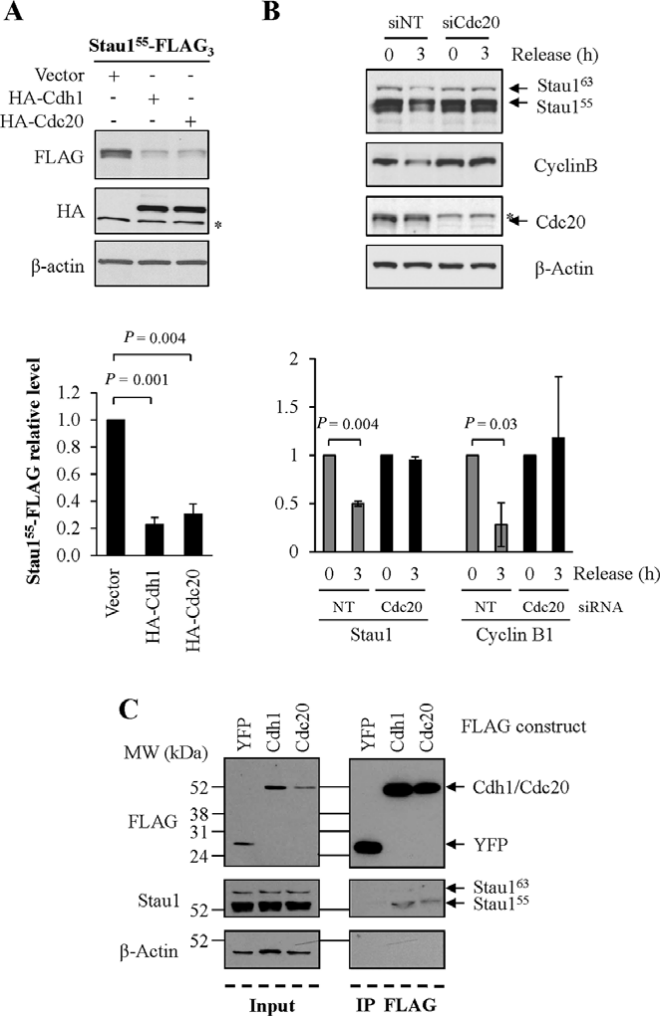
Stau1 down-regulation in mitosis is dependent on the APC/C. **(A)** HEK293T cells were co-transfected with plasmids coding for Stau1^55^-FLAG_3_ and HA-Cdc20, HA-Cdh1 or the empty vector as control. Cells extracts were analyzed by western blotting. *, unspecific signal. **(B)** HCT116 cells were transfected with siRNAs control or targeting the Cdc20 mRNA. Cells were synchronized in late G2 with RO-3306 (0) and released from the block for 3 h (3). Cell extracts were analyzed by western blotting. In A and B, the ratios between Stau1^55^-FLAG and β-Actin levels and the statistical analyses were calculated as in the legend of Figure 1, the ratio observed in cells transfected with the empty vector being arbitrarily set to 1. **(C)** HEK293T cells were transfected with plasmids coding for FLAG-Cdh1, FLAG-Cdc20 or FLAG-YFP as indicated. Left: input. Right: FLAG-tagged proteins were immunoprecipitated with anti-FLAG antibody and co-purified endogenous Stau1 was detected with anti-Stau1 antibody by western blotting. Each panel (A, B and C) is representative of three independently performed experiments.

The down-regulation profile of Stau1 during mitosis that coincides with that of known APC/C substrates (Figure 1A) and the observation that Stau1 can be degraded by the UPS suggest that it is a target of APC/C. Therefore, to determine whether APC/C^Cdc20^ and/or APC/C^Cdh1^ are involved in Stau1 destabilization, Stau1^55^-FLAG_3_ was expressed along with HA-tagged Cdh1 or Cdc20 to increase APC/C activity in asynchronous HEK293T cells. Figure 3A clearly showed that enforced expression of either Cdh1 or Cdc20 significantly reduced Stau1^55^-FLAG_3_ amounts indicating that Stau1^55^ is a novel substrate of these proteins. To fully establish their involvement in endogenous Stau1^55^ degradation, we down-regulated the expression of Cdc20 using siRNA and analyzed the levels of Stau1 expression during mitosis. siRNA-transfected HCT116 cells were synchronized at the G_2_/M transition with the Cdk1 inhibitor RO-3306 (38) and then released from the block for 3 h. As seen in Figure 3B, endogenous Stau1 was not degraded at 3 h post-release when isolated from siRNA-Cdc20-transfected cells as compared to control cells.

Because Cdh1 and Cdc20 contribute to substrate recognition and recruitment to APC/C, we tested whether Stau1^55^ can associate with either Cdh1 or Cdc20. HEK293T cells were transfected with FLAG-YFP, FLAG-Cdh1 or FLAG-Cdc20 expressors. FLAG-tagged proteins were purified and co-immunoprecipitated proteins were analyzed. Endogenous Stau1^55^ was detected in FLAG-Cdh1 and in FLAG-Cdc20 immune complexes (Figure 3C) whereas it was absent in control conditions, showing specific Stau1^55^/Cdh1 and Stau1^55^/Cdc20 interactions. These results support the notion that Stau1^55^ is a substrate of APC/C^Cdh1^ and APC/C^Cdc20^.

### The N-terminal domain of Stau1^55^ is required for its Cdh1- and Cdc20-dependent down-regulation

**Figure 4.**
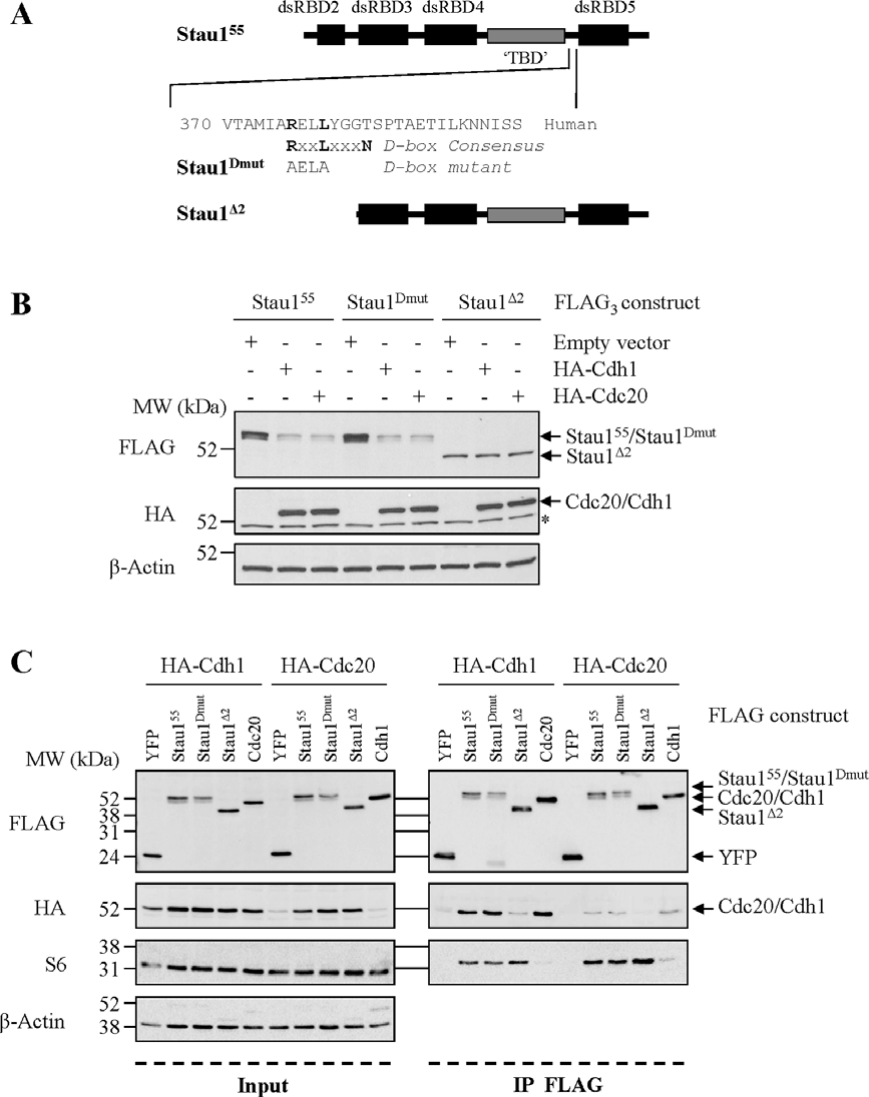
The N-terminal region of Stau1 is required for its down-regulation by APC/C. (**A**) The D-box and Stau1^Δ2^ mutated proteins are shown below the schematic representation of Stau1. dsRBD: double-stranded RNA-binding domain. TBD: tubulin-binding domain. (**B**) HEK293T cells were co-transfected with plasmids coding for Stau1^55^-FLAG_3_, Stau1^Dmut^-FLAG_3_ or Stau1^Δ2^-FLAG_3_ and HA-Cdc20, HA-Cdh1 or the empty vector as control. Cell extracts were analyzed by western blotting. *, unspecific signal. **(C)** HEK293T cells were co-transfected with plasmids coding for HA-Cdh1 or HA-Cdc20 and FLAG-YFP, Stau1^55^-FLAG, Stau1^Dmut^-FLAG, Stau1^Δ2^-FLAG, Cdc20-FLAG or Cdh1-FLAG, as indicated. Left: input. Right: FLAG-tagged proteins were immunoprecipitated with anti-FLAG antibody and co-purified proteins were detected with anti-HA and anti-S6 antibodies by western blotting. Anti-ribosomal protein S6 was used as control. Cdc20 being a known target of Cdh1, this interaction can be viewed as the positive control. Interaction with YFP is the negative control. Each panel (A, B and C) is the representative of three independently performed experiments.

APC/C^Cdc20^ and APC/C^Cdh1^ usually recognizes substrates containing D-box and/or KEN-box consensus sequences (31–33). A search for these motifs revealed the presence of a conserved D-box motif in the C-terminal half of Stau1 (Figure 4A). To test its functionality, the consensus sequence was mutated to generate Stau1^Dmut^ (Figure 4A). Then, HEK293T cells were co-transfected with plasmids coding for the wild-type Stau1^55^-FLAG_3_ or the D-box mutant protein and either HA-Cdc20 or HA-Cdh1 as described in Figure 3A. Our results indicated that Stau1^Dmut^-FLAG_3_, as observed with the wild-type Stau1^55^-FLAG_3_, was sensitive to Cdh1- and Cdc20-dependent destabilization (Figure 4B), suggesting that the putative D-box is not functional. Consistently, Stau1^Dmut^-FLAG_3_, as did Stau1^55^-FLAG_3_, interacted with Cdh1 and Cdc20 as it was detected in HA-Cdh1 and in HA-Cdc20 immune complexes in a co-immunoprecipitation experiment (Figure 4C).

As a means to identify a functional domain in Stau1^55^ that confers Cdh1- and Cdc20-mediated degradation, we immunoprecipitated HA-Cdc20 and HA-Cdh1 complexes from cells that co-expressed a series of Stau1 deletion mutants. While deletion of most Stau1^55^ domains did not impair Stau1 association with Cdh1 and Cdc20 (not shown), deletion of the first 88 N-terminal amino acids of Stau1^55^ (mutant Stau1^Δ2^) prevented it (Figure 4C). Anti-ribosomal protein S6 was used as a positive control since ribosomes are known to co-immunoprecipitate with Stau1 (25). To determine if the deletion also has an impact on Stau1^55^ stability, we co-transfected HEK293T cells with plasmids coding for Stau1^Δ2^-FLAG_3_ and either HA-Cdc20 or HA-Cdh1. Stau1^Δ2^-FLAG_3_ was protected from the Cdh1- and Cdc20-dependent destabilization (Figure 4B), indicating that the first 88 N-terminal amino acids of Stau1^55^ contributed to Stau1 degradation.

### Deregulation of Stau1 expression impairs mitosis entry

**Figure 5.**
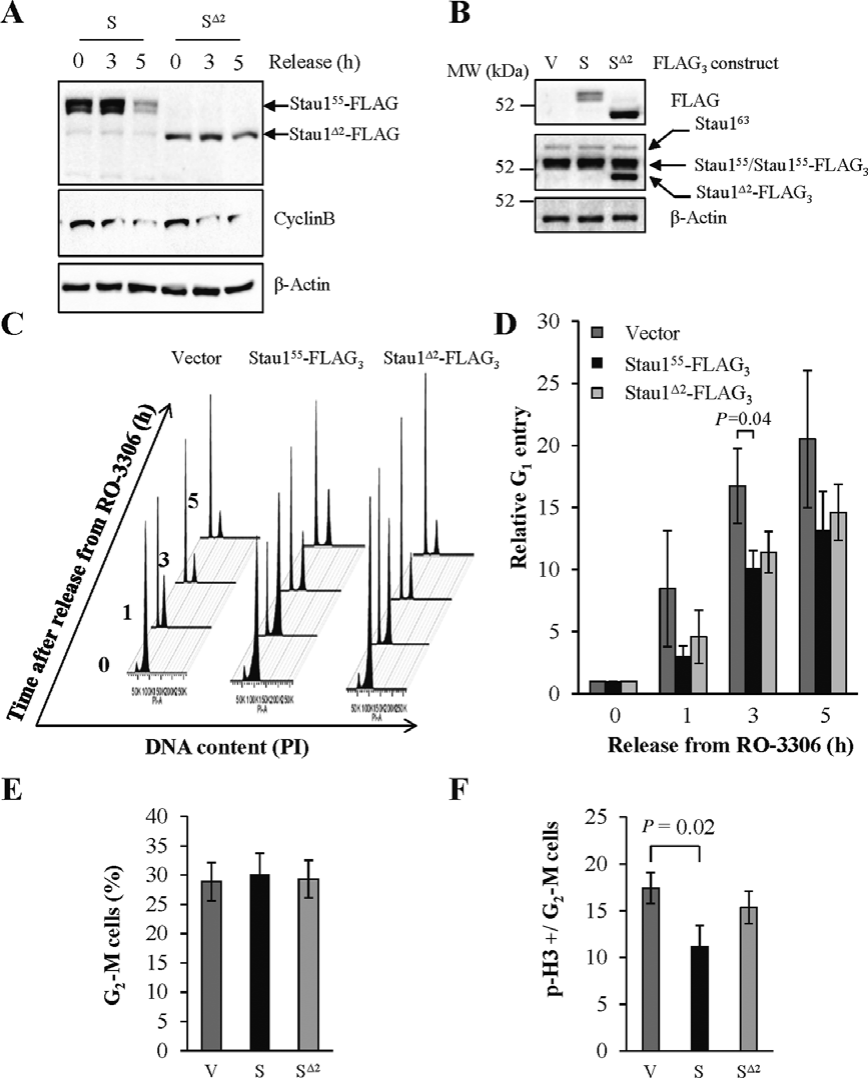
Expression of Stau1^55^-FLAG_3_ impairs mitosis entry. (**A**) HCT116 cells were infected with viruses expressing Stau1^55^-FLAG_3_ (S) or Stau1^Δ2^-FLAG_3_ (S^Δ2^), then synchronized at the G2/M transition with RO-3306 at 10 μM (0) and released for 3 h (3) or 5 h (5). Proteins in cell extracts were analyzed by western blotting. (**B**) HCT116 cells were infected with viruses expressing Stau1^55^-FLAG_3_, Stau1^Δ2^-FLAG_3_ or the empty vector (V) and Stau1 expression in asynchronous cells was analyzed by western blotting. (C and D) HCT116 cells were infected as in B, synchronized at the G_2_/M phase transition with RO-3306 and then released from the block. At different time points post-release, cells were harvested and analyzed by FACS to calculate the percentage of cells in G_1_. A representative experiment (**C**) and the means of three independently performed experiments (**D**) are shown. The graph in D represents the mean and standard deviation of the ratio of the number of cells in the G_1_ phase at specific time post-release over that at time 0. Statistical analyses (Student's *t*-test) are indicated when significant. (**E**) Infected unsynchronized HCT116 cells were harvested, stained with Hoechst solution and analyzed by FACS to calculate the percentage of cells in the G_2_-M phases of the cell cycle. The statistical analysis (Student's *t*-test) of three independently performed experiments is provided. (**F**) Cells harvested in E were also stained with anti-phospho-histone H3 (p-H3) antibody to determine the relative amounts of mitotic cells within the G_2_-M population. The ratio of the percentage of p-H3 positive cells on G_2_-M cells is shown. The statistical analysis (Student's *t*-test) of three independently performed experiments is provided.

To determine if the stabilization of Stau1^Δ2^-FLAG_3_ occurs during the cell cycle, HCT116 cells were infected with viruses expressing Stau1^55^-FLAG_3_ or Stau1^Δ2^-FLAG_3_, synchronized at the G_2_/M transition with RO-3306 and released from the block for 3 or 5 h. As expected, Stau1^55^-FLAG_3_ was degraded during mitosis whereas Stau1^Δ2^-FLAG_3_ was not (Figure 5A).

To understand the biological relevance of APC/C-mediated Stau1 decline and determine whether Stau1^55^ stabilization is detrimental for mitosis, the impact of Cdh1/Cdc20-resistant Stau1^Δ2^-HA_3_ expression was studied. HCT116 cells were infected with viruses expressing the empty vector, Stau1^55^-FLAG_3_ or Stau1^Δ2^-FLAG_3_. Infected cells were synchronized at the G_2_/M phase border with RO-3306 for 20 h and the block was released for different time periods. Western blotting indicated that expression of Stau1^55^-FLAG_3_ and Stau1^Δ2^-FLAG_3_ was below that of the endogenous Stau1^55^ and expression of Stau1^Δ2^-FLAG_3_ was higher than that of Stau1^55^-FLAG_3_ (Figure 5B). At each time point, cells were collected and analyzed by FACS to determine the percentage of cells in G_1_, as a means to compare the time it took to transit through mitosis and enter the G_1_ phase. Expression of Stau1^55^-FLAG_3_ caused a delay in G_1_ entry as observed in three independently performed experiments (Figure 5C). Indeed, at 3 h post-release, the number of cells in G_1_ was significantly reduced in Stau1^55^-FLAG_3_-expressing cells as compared to control cells (Figure 5D). A delay in G_1_ entry was also observed in Stau1^Δ2^-FLAG_3_-expressing cells although it was not significant (*P*-value = 0.07) as compared to cells infected with the empty vector (Figure 5D).

To expand upon these results, we compared the percentage of cells in each phase of the cell cycle. We hypothesized that if the length of mitosis is increased in Stau1^55^-FLAG_3_-expressing cells, the percentage of cells in this phase should be greater in these cells than in vector-infected cells. Asynchronous cells were collected 2 days post-infection and analyzed by FACS. Our data revealed that the percentage of cells in each phase of the cell cycle was quite similar for the three conditions (Figure 5E). However, it is possible that the number of cells in the G_2_ phase (which is much longer than mitosis) masked a relatively small difference in the number of mitotic cells in asynchronous cells. To more specifically study cells in mitosis, we labeled infected cells with an antibody recognizing phospho-histone H3 (Ser10) before analysis by FACS. Our data indicated that the percentage of phospho-H3 positive cells is decreased in Stau1^55^-FLAG_3_-infected cells as compared to control cells (Figure 5F), suggesting that fluctuation of Stau1 level may impair the G_2_/M transition. In contrast, expression of Stau1^Δ2^-FLAG_3_ did not significantly change the percentage of mitotic cells as compared to control cells. Altogether, these results indicate that the levels of expression of Stau1 during the G_2_-M phases of the cell cycle influence the timing of mitosis and that the putative molecular mechanism that regulates mitosis entry involves the N-terminal domain of Stau1.

### Stau1^55^-FLAG_3_ expression impairs cell proliferation in transformed cell lines

**Figure 6.**
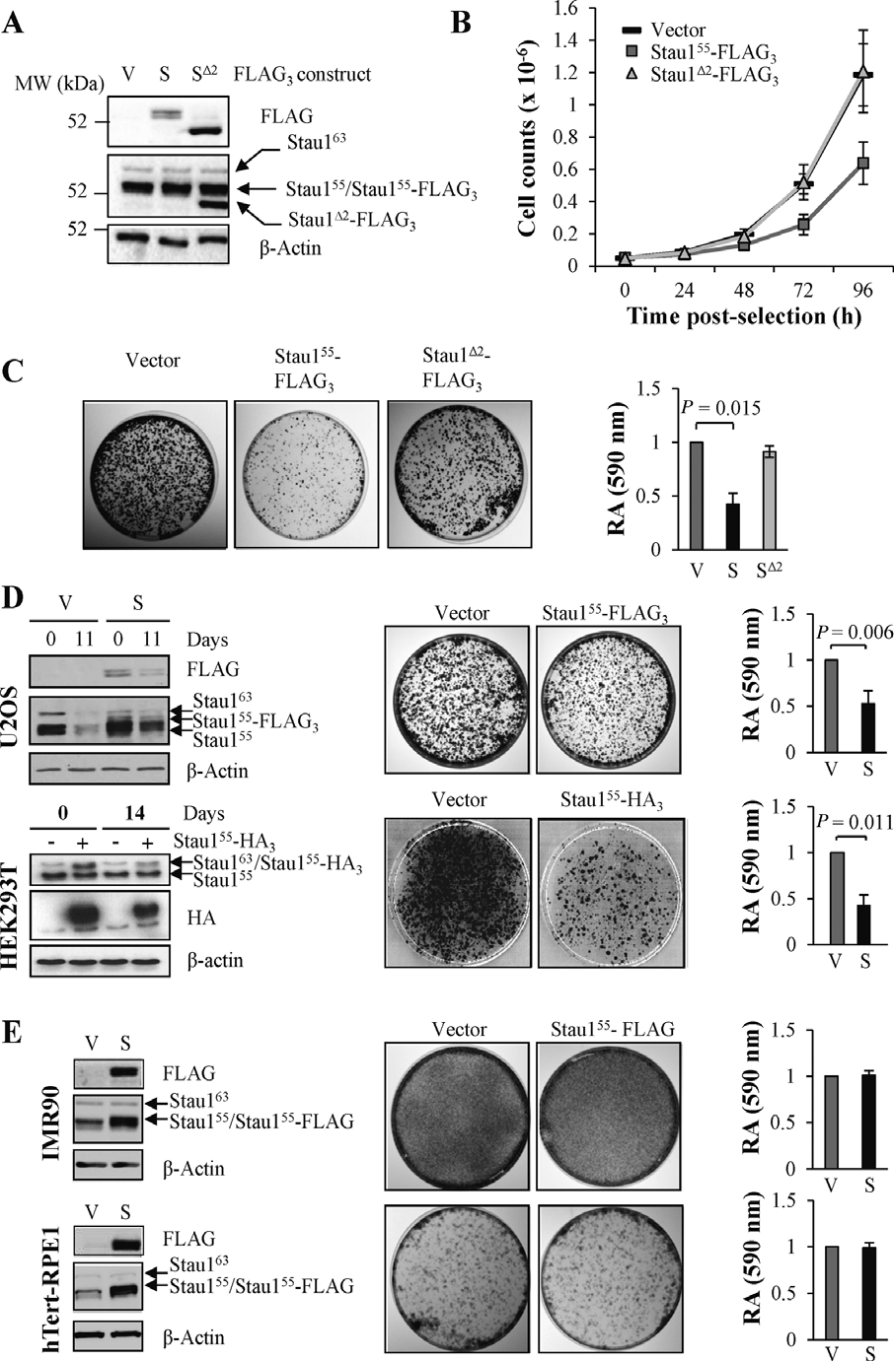
Stau1^55^-FLAG_3_ expression impairs cell proliferation. (**A**) HCT116 cells were infected with viruses expressing Stau1^55^-FLAG_3_, Stau1^Δ2^-FLAG_3_ or the empty vector and Stau1 overexpression was analyzed by western blotting. (**B**) HCT116 infected cells were grown in the presence of puromycin for 2 days and then replated at the same density in fresh medium. Cells were harvested every day and the number of cells was counted using a hemacytometer. The graph shows the means and standard deviation of cell counts of three independently performed experiments. (**C**) HCT116 cells were plated as in (B). Colony formation assays were performed to measure cell proliferation in cells expressing Stau1^55^-FLAG_3_, Stau1^Δ2^-FLAG_3_ or the empty vector when grown for 10 or 14 days. Results are representative of three independently performed experiments. Colonies were stained with crystal violet. The stain was extracted and quantified by measuring absorbance at 590 nm. Quantification of the relative amounts of stain in cells transfected with the empty vector, Stau1^55^-FLAG or Stau1^Δ2^-FLAG_3_ is expressed as the mean and standard deviation of three independent experiments, absorbance of cells infected with the empty vector being arbitrarily fixed to 1. The statistical analysis (Student's *t*-test) is provided. V, empty vector; S, Stau1^55^-FLAG_3_; S^Δ2^, Stau1^Δ2^-FLAG_3_. (**D**) Transformed cell lines (U2OS and HEK293T) and (**E**) untransformed cell lines (IMR90 and htert-RPE1) were infected with viruses expressing the empty vector (V) or Stau1^55^-FLAG_3_ or Stau1^55^-HA_3_ (S) as indicated and plated at the same density for the colony formation assay. Left: western blot analyses of cells infected with the indicated viruses. Middle: representative data of three independently performed experiments. Right: quantification of crystal violet stained cells as described above.

To study the consequence of Stau1 level fluctuation on successive cell divisions, the impact of Stau1^55^-FLAG_3_ and Stau1^Δ2^-FLAG_3_ expression on cell cycle proliferation was first assessed by growth curve assays. Expression of Stau1^55^-FLAG_3_ and Stau1^Δ2^-FLAG_3_ at day 0 was below that of the endogenous Stau1^55^ (Figure 6A). Consistent with the mitotic effect described above (Figure 5), expression of Stau1^55^-FLAG_3_ clearly caused a proliferative defect (Figure 6B). In contrast, expression of Stau1^Δ2^-FLAG_3_ had no visible deleterious effect on cell proliferation (Figure 6B).

The long-term effect of Stau1^55^-FLAG_3_ and Stau1^Δ2^-FLAG_3_ expression was analyzed using a colony formation assay. Consistent with the growth curve experiments, our data indicated that Stau1^55^-FLAG_3_ expressing cells formed fewer/smaller colonies. In contrast, Stau1^Δ2^-FLAG_3_ expressing cells grew essentially as efficiently as cells infected with the empty vector (Figure 6C). We further showed that expression of Stau1^55^-FLAG_3_ or Stau1^55^-HA_3_ also impaired cell proliferation in U2OS and HEK293T cells, respectively (Figure 6D). In contrast, Stau1^55^-FLAG_3_ expression had no impact on cell proliferation when the colony formation assay was repeated in two non-transformed human cell lines, the hTERT-immortalized retinal pigment epithelial cell line htert-RPE1 and the human fetal lung fibroblast IMR90 cells (Figure 6E). These results support the idea that deregulation of Stau1 protein levels is deleterious for proliferation of transformed cells and that the N-terminal domain is required for this function.

We next determined if the knockdown of Stau1 expression had a similar detrimental effect on cell proliferation. HCT116 and HEK293T cells were infected with viruses expressing either one of two different shRNAs that targeted Stau1 or a non-targeted (NT) shRNA. Western blot analyses indicated that the efficacy of Stau1 knock-down was constant throughout the experiments (data not shown). In these conditions, both infected NT- and Stau1-KD-cells had significantly the same growth rate and numbers of colonies (data not shown) indicating that Stau1 expression is not essential for cell proliferation in transformed cell lines.

### Stau1^55^-FLAG_3_ expression does not induce cell death or cell cycle exit

To determine if the observed decrease in the number of proliferative cells is a consequence of cell death caused by Stau1^55^-FLAG_3_ expression, we first looked for signs of apoptotic cells at day four post-infection when cells showed impaired proliferation. Apoptosis was analyzed using anti-PARP1 antibody since, in apoptotic cells, PARP1 is cleaved to generate a degradation product of 89 kDa. In these conditions, there was no sign of PARP1 cleavage (Supplementary Figure S2A), indicating that apoptotic cells are not present in the cultures of Stau1^55^-FLAG_3_-expressing cells. Similarly, at day 4 post-infection, the number of quiescent or senescent cells in the Stau1^55^-FLAG_3_- and Stau1^Δ2^-FLAG_3_-expressing cultures was not significantly different from that observed in the control cells (Supplementary Figure S2B and C).

### Microarray analyses of mitotic Stau1^55^-bound mRNAs

**Figure 7.**
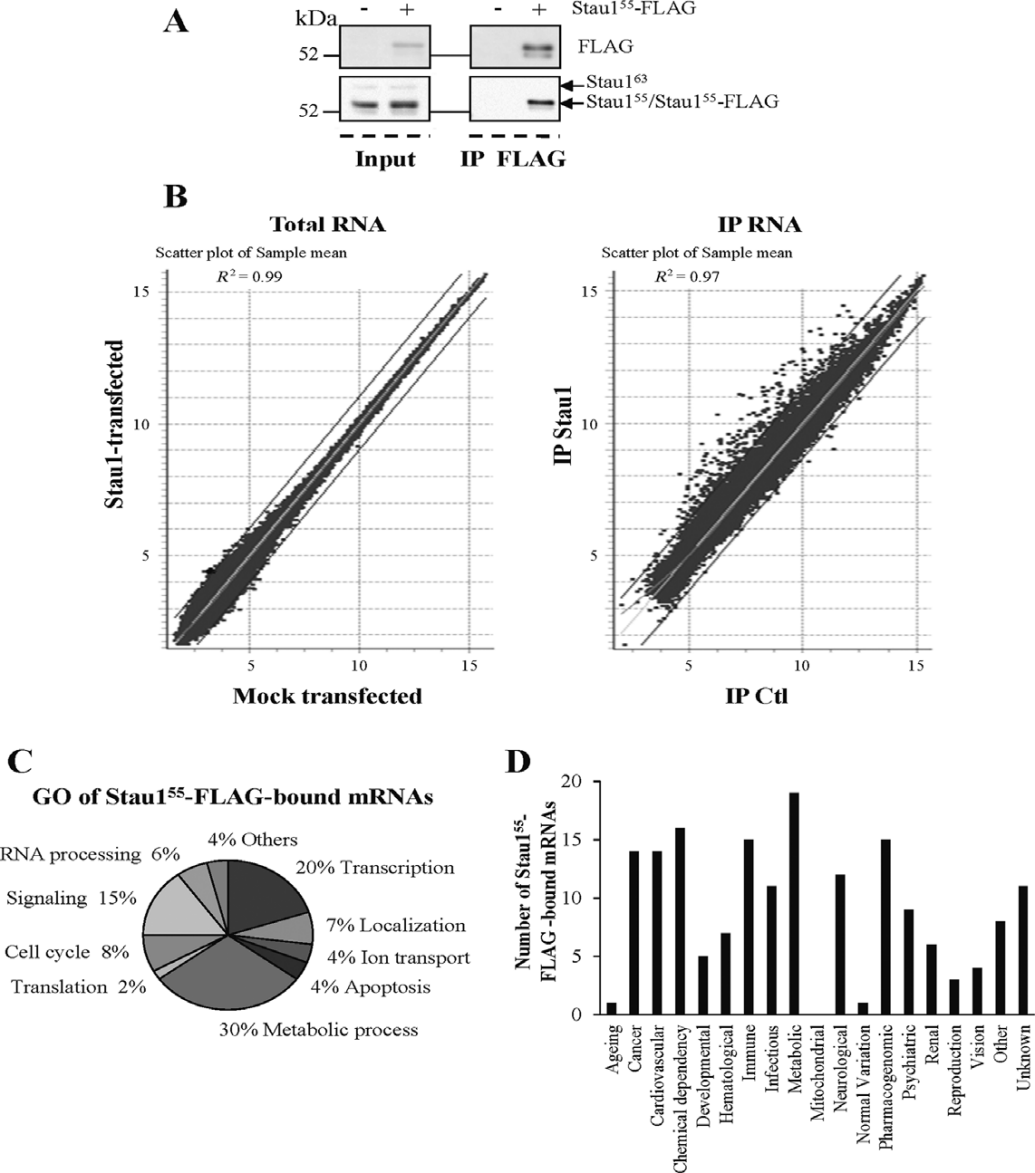
Microarray analyses of Stau1-transfected HEK293T cells in prometaphase. (**A**) HEK293T cells were transfected with plasmids coding for the empty vector or Stau1^55^-FLAG and then synchronized with nocodazole. Prometaphase cells were collected and protein levels were monitored by western blotting (input). Cell extracts were also used to immunoprecipitate Stau1^55^-FLAG and its bound mRNAs. Proteins were analyzed by western blotting as above (after IP). (**B**) Total RNA and RNAs isolated in Stau1^55^-FLAG immune complexes of prometaphase cells were used to hybridize microarrays (*n* = 3). Scatter plots (as a means to investigate a possible relationship between two sets of data) are shown. (**C**) Stau1^55^-FLAG-bound mRNAs were identified by microarray hybridization (*n* = 3) and grouped according to the gene ontology database. (**D**) Stau1^55^-FLAG-bound mRNAs were analyzed with the Genetic Association Database program to identify those that are associated with diseases.

Our results argue for an important regulation of Stau1 levels during the G_2_ and/or M phases of the cell cycle. As a major post-transcriptional regulator, Stau1 may exert its role(s) through the spatial and/or temporal regulation of its bound mRNAs. Therefore, to gather clues to support this possibility, we first determined if Stau1^55^-FLAG expression significantly modified the transcriptome of transformed cells in prometaphase explaining the observed impairment in mitosis transit and/or cell proliferation. To this end, we transfected HEK293T cells with plasmids coding for Stau1^55^-FLAG or the empty vector, synchronized the cells in prometaphase and compared the level of expression of mRNAs in these cells. As seen in Figure 7A, the level of the FLAG protein was less than that of the endogenous Stau1. Using microarray hybridization, we showed that only four probes were up- or down-regulated more than 2-fold (*P*-value < 0.05) in Stau1^55^-FLAG-transfected cells as compared to vector-transfected cells (Figure 7B and Supplementary Tables S1 and S2). Our data indicate that the transcriptome of the cells in prometaphase is not largely modified by Stau1^55^-FLAG expression. This is consistent with a recent transcriptome study that concludes that varying Stau1 intracellular concentration has an extremely subtle effect on mRNA levels (10).

Second, we identified Stau1-bound mRNAs in prometaphase as a means to identify mRNAs that could be modulated in response to Stau1 differential expression. Using cell extracts prepared from Stau1- and vector-transfected cells as controls, Stau1^55^-FLAG was immunoprecipitated using anti-FLAG antibody. Stau1^55^-FLAG-bound mRNAs were purified and used to hybridize human microarrays. A total of 275 transcripts were enriched at least 2.5-fold in immune-complexes isolated from Stau1^55^-FLAG-expressing cells as compared to those isolated from vector-transfected cells (Figure 7B and Supplementary Tables S3 and S4), 91 of which containing inverted Alu sequences in their 3′UTR. When each probe was assigned to a GO term, the most frequent terms were related to metabolic processes, transcription and signaling (Figure 7C and Supplementary Table S5). Interestingly, 8% of the probes coded for proteins involved in cell cycle. Accordingly, when analyzed with the Genetic Association Database program, 19 and 14 of the Stau1-bound mRNAs were linked to metabolic diseases and cancer, respectively (Figure 7D). When analyzed with the Database for Annotation, Visualization and Integrated Discovery (DAVID) functional annotation tool (42) to assign cellular functions to mRNAs that were enriched in Stau1^55^-containing ribonucleoproteins (RNPs), the most prevalent terms were related to zinc finger domain and p53 signaling pathway (Supplementary Tables S6 and S7).

**Figure 8.**
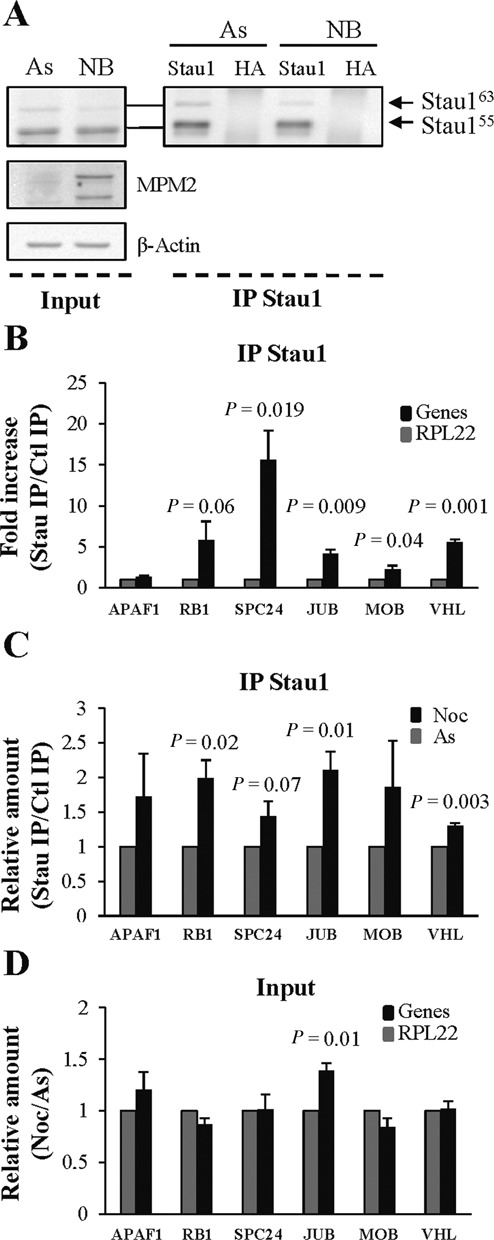
RT-qPCR validation of microarray data using selected mRNAs. (**A**) HEK293T cells were left asynchronous (As) or synchronized in prometaphase with nocodazole (Noc) (input). Endogenous Stau1-containing mRNPs were immunopurified using anti-Stau1 antibody (after IP). Anti-HA antibody was used as control. Proteins were visualized by western blotting. (**B**) Endogenous Stau1-containing mRNPs were immunopurified from nocodazole-treated cells using anti-Stau1 antibody. Anti-HA antibody was used as control. Co-immunoprecipitated mRNAs were isolated and RT-qPCR amplified with specific primers. The ratios of gene expression for each transcript in Stau1-IP versus control-IP were normalized to that of RPL22 used as control. (**C**) Endogenous Stau1-containing mRNPs were immunopurified from asynchronous and nocodazole-treated cells as in B. Co-immunoprecipitated mRNAs were isolated and RT-qPCR amplified with specific primers. The ratios of gene expression for each transcript in Stau1-IP versus control-IP normalized to that of RPL22 were calculated in asynchronous (As) and prometaphase (Noc) cells. The ratio in asynchronous cells was arbitrary fixed at 1. (**D**) Total mRNA was isolated from asynchronous and nocodazole-treated cells and the expression of the six transcripts was quantified by RT-qPCR. The ratios of gene expression in nocodazole-treated cells versus asynchronous cells were normalized to that of RPL22. Each panel (B, C and D) represents the means and standard deviations of three independently performed experiments. The statistical analysis (Student's *t*-test) is provided. RPL = RPL22, ribosomal protein L22; APAF1, apoptotic peptidase activating factor 1; RB1, retinoblastoma 1; SPC24, NDC80 kinetochore complex component; Jub, ajuba homolog; MOB = MOBKL2B, Mps One Binder kinase activator-like 2B; VHL, von Hippel-Lindau tumor suppressor.

To complement the microarray data, we further studied six transcripts based on the role of their encoding protein in the cell cycle. These RNAs were enriched at least 2-fold in Stau1-containing complexes as compared to controls in prometaphase (Supplementary Table S4) or asynchronous (22) cells. Their expression and their association with endogenous Stau1 in prometaphase versus asynchronous HEK293T cells was studied. First, Stau1-bound mRNAs were co-immunoprecipitated from nocodazole-arrested cells using anti-Stau1 antibody and anti-HA antibody as control (Figure 8A). Quantification of mRNA amounts in the immunoprecipitates was performed by RT-qPCR. A ratio between the amount of mRNAs in Stau1-IPs and control-IPs was calculated. The resulting ratios were then normalized over that of RPL22 mRNA, an abundant transcript not associated with Stau1 according to the microarray data. Five of the six studied mRNAs showed an increase of at least 2-fold in the Stau1-IP/control-IPs ratios as compared to that of RPL22 (Figure 8B), confirming the microarray results. In addition, all six mRNAs were enriched between 2.2 and 13.5 times in Stau1-IP complexes as compared to total RNA extracts (input). Then, we compared the amount of Stau1-bound mRNAs in prometaphase cells to that in asynchronous cells. Our data showed an increase of each mRNA in Stau1-containing complexes purified from nocodazole-treated versus asynchronous cells (Figure 8C). This increase was not simply due to enhanced steady state levels of these mRNAs in total cell extracts of nocodazole-treated cells as compared to asynchronous cells. Indeed, our data indicated that the expression of the six genes was quite similar in both cell conditions (Figure 8D), except for Ajuba (JUB) mRNA that showed a significant 1.38-fold increase in the ratio on mRNAs in prometaphase versus asynchronous cells as compared to that of RPL22.

## DISCUSSION

The periodic expression of cell cycle regulatory proteins is a consequence of their controlled synthesis coupled to their targeted proteolysis. It is well established that transcription factors and ubiquitin ligases play predominant roles in this event (26–28). Although post-transcriptional regulation contributes to the fine tuning of gene expression (1), the involvement of RNA-binding proteins in the control of cell cycle progression is less appreciated. In this paper, we show that the levels of the multifunctional RNA-binding protein Stau1 fluctuate during the cell cycle and describe a novel mechanism by which Cdh1 and Cdc20, two co-activators of APC/C, control Stau1 abundance. The relevance of this phenomenon during the cell cycle is illustrated by the observation that overexpression of Stau1 affects mitosis entry and impairs cell proliferation. These results raise the interesting possibilities that Stau1 is an important post-transcriptional regulator of genes involved in cell cycle progression and that APC/C^Cdh1^ and APC/C^Cdc20^ control the fate of relevant mRNAs via the modulation of Stau1 levels.

### The APC/C co-activators Cdh1 and Cdc20 are major regulators of Stau1 stability

As expected from the pattern of Stau1 cell cycle-dependent decay that mirrors the period of activity of APC/C, we show that this enzymatic complex is involved in Stau1 degradation. It is likely that its activity on Stau1 is mediated by Cdh1 and Cdc20 since their overexpression leads to a decrease of Stau1 abundance and since Cdc20 down-regulation stabilizes Stau1 levels in mitosis. Moreover, a functional domain was identified in the N-terminus of Stau1 that allows Stau1 to interact with Cdh1 and Cdc20. All these results, combined with the observation that Stau1 is degraded by the UPS, strongly support the idea that Stau1 is a target of the E3 ubiquitin ligase APC/C.

In contrast to several APC/C targets, only a partial degradation of Stau1 was observed following mitosis exit even if APC/C^Cdh1^ activity persists in early G_1_ (34,43–47). This partial down-regulation of Stau1 was also observed in a high throughput analysis that reported a 2-fold reduction of Stau1 protein level in G_1_ cells as compared to that in prometaphase-arrested cells (48). These results suggest that a subpopulation of Stau1 is protected from Cdh1-dependent destabilization. Since phosphorylation of some of the APC/C^Cdh1^ substrates was shown to prevent their degradation (49–52), a similar mechanism may limit the accessibility of APC/C to Stau1. Alternatively, a differential subcellular distribution of a fraction of Stau1 could prevent its decay. Since Stau1 is a multifunctional protein, its partial and/or spatial degradation may contribute to shut-down one or some of its functions no longer required or deleterious for cell cycle progression while allowing it to execute other tasks important for different cell processes. Our results suggest that the Stau1 sub-population that is degraded in mitosis is associated with mRNAs coding for cell cycle-related proteins since these transcripts are more abundant in Stau1-immunoprecipitates prepared from prometaphase cells than those from asynchronous cells (Figure 8C).

### The Stau1 N-terminal region impairs cell cycle progression

APC/C-dependent substrate degradation usually relies on the presence of a D-box or KEN-box recognition motif. Our results indicate that the putative D-box motif found at the C-terminus of Stau1 is not required for Cdh1 and Cdc20 binding or for Stau1 degradation (Figure 4), suggesting that a non-classical motif may play a similar role. This motif lies within the first N-terminal 88 amino acids of Stau1. No known APC/C recognition motif is predicted in this region. Strangely, although this protein is not degraded in mitosis, its expression has no effect on cell proliferation. It is rather the expression of the full length protein that delays mitosis entry and reduces cell proliferation. It is likely that, in addition to its role in APC/C association, the N-terminus of wild-type Stau1^55^ plays additional roles related to inhibition of cell proliferation. Identification of the precise Cdh1/Cdc20-binding site and mutation of the motif by point mutations will be necessary to specifically address the relevance of Stau1 degradation in mitosis. It is still unknown if all these phenotypes (Cdh1 binding, Cdc20 binding, impairment of mitosis entry and inhibition of cell cycle progression) are carried out by a unique motif or if they require multiple determinants. The molecular function of the N-terminal domain is still unclear. Although its sequence is similar to the consensus sequence of the C-terminal half of a double-stranded RNA-binding domain, no RNA-binding activity was associated to it (8). In Drosophila, the homologous domain was shown to be involved in mRNA transport/localization (53). In mammals, such a role for this domain has not been documented so far. Indeed, previous studies excluded this region from being involved in RNA binding (7,8), translation (3), nuclear import (54), sub-cellular localization (25,54) and UPF1 association for Staufen1-mediated mRNA decay (SMD) (6). All these functions were mapped to regions located outside the N-terminus of Stau1. Nevertheless, although Stau1 dimerization was shown to mainly involve dsRBD3 and dsRBD5 (55,56), a weak contribution of dsRBD2 was also reported (55). In addition, the N-terminus of Stau1 was shown to promote Pr55^Gag^–Pr55^Gag^ interaction in the course of HIV-1 assembly (41). However, none of these studies were done during mitosis. We do not exclude the possibility that, as cells divide, the N-terminal region of Stau1 may be tagged for example by post-translational modifications and/or be associated with specific cofactors to modulate one or several activities of the protein.

### Biological relevance of Stau1 regulation for cell cycle progression and post-transcriptional regulation

Our results indicate that Stau1 level during the cell cycle is reminiscent of that of other APC/C substrates. As most of them play important roles in mitosis (57), it was consistent to observe that fluctuation of Stau1 level impairs mitosis. Indeed, effects on mitosis were described following overexpression of other APC/C substrates like the Polo-like kinase 1 (Plk1) and the mitotic spindle-associated proteins TMAP/CKAP2 and NuSAP (58–61). In these cases, impaired cytokinesis and structural defects in the mitotic spindle were shown to be the major factors for mitosis delay or cell cycle arrest. Therefore, one interpretation of our results is that Stau1 has mitotic functions that must eventually be inhibited for proper progression through mitosis.

In addition to mitosis, Stau1^55^-FLAG_3_ expression also impaired cell proliferation. Our results are in agreement with those of a large-scale screen in which Stau1 significantly inhibited colony formation when transfected in human hepatoma cells (62). It is possible that cell proliferation impairment associated with Stau1 overexpression is a result of the accumulation of the observed mitotic delay over multiple rounds of cell division. For example, it was shown that a partial disassembly of the mitotic apparatus did not affect the current division but prevented the formation of a functional spindle during the next mitosis (63). Similarly, a slight overexpression of a non-degradable form of the cytoskeleton-associated protein 2 (CKAP2), a target of APC/C, does not prevent the cell to go through the process of cell division. However, abnormalities in spindle assembly appear during the subsequent mitosis (58). Although it is not known whether Stau1 participates in spindle functions, this possibility would be consistent with the fact that Stau1^55^-FLAG_3_ expression causes a relatively weak delay in mitosis and a stronger impairment of cell proliferation. Nevertheless, we do not exclude the possibility that these two phenotypes involve different mechanisms and/or perturb different phases of the cell cycle. Although the mechanism is not yet defined, our studies excluded apoptosis, senescence and quiescence as major factors for the observed decreased cell proliferation.

Strikingly, the phenotypes due to Stau1 overexpression are observed in three cancer cell lines but not in two untransformed cells. Although it may be too early to generalize to all cancers and untransformed cells, our data suggest that Stau1 may restore, at least partly, some pathways that had been modified and/or overcome to allow unregulated proliferation of cancer cells. Based on the fact that Stau1^55^-FLAG_3_ expression restrains mitosis entry, Stau1 may be somehow involved in mechanisms that lead to checkpoint decisions in G_2_ and/or at the G_2_/M transition. Such a major role for Stau1 during the G_2_ and G_2_/M phases of the cell cycle is also consistent with its pattern of expression through the cell cycle which reveals a peak of expression during this time period (see also below). Thus, its overexpression in transformed cells could result in the reinforcement of the mechanisms of checkpoint regulation that are often lost in genetically unstable cancer cells. If involved in mechanisms of regulation, it is not unexpected that Stau1 down-regulation has no effect on proliferation of cancer cells that have modified and optimized each pathway for unregulated cell division.

Stau1-bound mRNAs isolated in prometaphase partly overlap those in asynchronous cells. Although comparisons are sometimes hard to interpret since experiments from different laboratories were done and analyzed by different techniques, almost half of the genes found in this study as Stau1 targets in prometaphase had previously been reported in other independent studies (10,11,22), confirming the specificity of our approach. The other half of the transcripts may be prometaphase-enriched targets since all other studies were done in asynchronous cells. It would be consistent with data in Figure 8C that shows that specific Stau1-bound mRNAs are more abundant in the Stau1-immunoprecipitates from cells in prometaphase than in asynchronous cells. Interestingly, 33% of the Stau1-bound mRNAs in prometaphase cells contain opposite-polarity Alu elements in the 3′UTR, a percentage that is quite similar to those reported in interphase cells (10,11).

Stau1 is a multifunctional protein involved in the post-transcriptional regulation of gene expression including mRNA transport, translation, decay, nuclear export and splicing. It is thus difficult to predict its precise involvement in the processes of cell division. However, based on recent studies (10,11,13), specific hypotheses can be proposed. It was shown that overexpression of transfected Stau1 increases ribosome occupancy and thus translation of bound mRNAs (10,11). In our study, we demonstrate that the amount of endogenous Stau1 is at least 2-fold higher in the G_2_ phase of the cell cycle than in G_1_, suggesting that Stau1-bound mRNAs may be translated more efficiently during G_2_ than G_1_. As Stau1 binds many mRNAs coding for zinc-finger proteins involved in transcriptional and post-transcriptional mechanisms (Supplementary Tables S6 and S7), the temporal increase expression of Stau1 would trigger a cascade that is progressively amplified through post-transcriptional and transcriptional effects that will ultimately modify the expression of multiple genes. Its subsequent degradation by the APC/C would silence these mRNAs whose expression may no longer be required as cells exit mitosis.

However, not all Stau1-bound mRNAs are subjected to enhanced translation upon Stau1 overexpression (10). Therefore, Stau1 may play a different role on these mRNAs. Several studies have shown that specific mRNAs are localized on the mitotic spindles in Drosophila, Xenopus and the human HeLa cell line (64–67). It is noteworthy that 6% of the Stau1-bound mRNAs in prometaphase cells are among those that are enriched on the mitotic spindle of human cells (65). Thus, one attractive possibility is that Stau1 participates in the localization, sequestration and/or anchoring of these mRNAs on the mitotic apparatus. This mechanism may ensure that both daughter cells receive equal amounts of these transcripts after cell division. The presence of a tubulin-binding domain in Stau1 protein and its involvement in the microtubule-dependent transport of mRNAs in neurons are consistent with this hypothesis (4,5,8). Through this mechanism, Stau1 could control a second mRNA regulon and its degradation in mitosis may trigger the release of its bound mRNAs and allow their timely translation for proper progression through the cell cycle.

Through the cell cycle-dependent proteolysis of numerous substrates, APC/C is required for cell survival and proliferation. The list of its known targets not only includes important cell cycle regulators such as cyclins, mitotic kinases and organizers of the cytoskeleton, but also modulators of gene expression like transcription factors and components of E3 ubiquitin ligases complexes (45,68–72). In Arabidopsis, APC/C also targets DRB4 (dsRNA-binding protein 4) a protein involved in RNA silencing (73). Our results with Stau1 add an RNA-binding protein to this growing list of APC/C effectors in mammals, suggesting that post-transcriptional mechanisms are also subject to APC/C control.

## SUPPLEMENTARY DATA

Supplementary Data are available at NAR Online.

SUPPLEMENTARY DATA

## References

[1] Keene J.D. (2007). RNA regulons: coordination of post-transcriptional events. Nat. Rev..

[2] Moore M.J. (2005). From birth to death: the complex lives of eukaryotic mRNAs. Science.

[3] Dugre-Brisson S., Elvira G., Boulay K., Chatel-Chaix L., Mouland A.J., DesGroseillers L. (2005). Interaction of Staufen1 with the 5′ end of mRNA facilitates translation of these RNAs. Nucleic Acids Res..

[4] Kanai Y., Dohmae N., Hirokawa N. (2004). Kinesin transports RNA: isolation and characterization of an RNA-transporting granule. Neuron.

[5] Kiebler M.A., Hemraj I., Verkade P., Kohrmann M., Fortes P., Marion R.M., Ortin J., Dotti C.G. (1999). The mammalian staufen protein localizes to the somatodendritic domain of cultured hippocampal neurons: implications for its involvement in mRNA transport. J. Neurosci..

[6] Kim Y.K., Furic L., Desgroseillers L., Maquat L.E. (2005). Mammalian Staufen1 recruits Upf1 to specific mRNA 3′UTRs so as to elicit mRNA decay. Cell.

[7] Marion R.M., Fortes P., Beloso A., Dotti C., Ortin J. (1999). A human sequence homologue of Staufen is an RNA-binding protein that is associated with polysomes and localizes to the rough endoplasmic reticulum. Mol. Cell. Biol..

[8] Wickham L., Duchaine T., Luo M., Nabi I.R., DesGroseillers L. (1999). Mammalian staufen is a double-stranded-RNA- and tubulin-binding protein which localizes to the rough endoplasmic reticulum. Mol. Cell. Biol..

[9] Vessey J.P., Macchi P., Stein J.M., Mikl M., Hawker K.N., Vogelsang P., Wieczorek K., Vendra G., Riefler J., Tubing F. (2008). A loss of function allele for murine Staufen1 leads to impairment of dendritic Staufen1-RNP delivery and dendritic spine morphogenesis. Proc. Natl. Acad. Sci. U.S.A..

[10] Ricci E.P., Kucukural A., Cenik C., Mercier B.C., Singh G., Heyer E.E., Ashar-Patel A., Peng L., Moore M.J. (2014). Staufen1 senses overall transcript secondary structure to regulate translation. Nat. Struct. Mol. Biol..

[11] de Lucas S., Oliveros J.C., Chagoyen M., Ortin J. (2014). Functional signature for the recognition of specific target mRNAs by human Staufen1 protein. Nucleic Acids Res..

[12] Kim Y.K., Furic L., Parisien M., Major F., DesGroseillers L., Maquat L.E. (2007). Staufen1 regulates diverse classes of mammalian transcripts. EMBO J..

[13] Elbarbary R.A., Li W., Tian B., Maquat L.E. (2013). STAU1 binding 3′ UTR IRAlus complements nuclear retention to protect cells from PKR-mediated translational shutdown. Genes Dev..

[14] Ravel-Chapuis A., Belanger G., Yadava R.S., Mahadevan M.S., DesGroseillers L., Cote J., Jasmin B.J. (2012). The RNA-binding protein Staufen1 is increased in DM1 skeletal muscle and promotes alternative pre-mRNA splicing. J. Cell Biol..

[15] Belanger G., Stocksley M.A., Vandromme M., Schaeffer L., Furic L., DesGroseillers L., Jasmin B.J. (2003). Localization of the RNA-binding proteins Staufen1 and Staufen2 at the mammalian neuromuscular junction. J. Neurochem..

[16] Gautrey H., McConnell J., Lako M., Hall J., Hesketh J. (2008). Staufen1 is expressed in preimplantation mouse embryos and is required for embryonic stem cell differentiation. Biochim. Biophys. Acta.

[17] Yamaguchi Y., Oohinata R., Naiki T., Irie K. (2008). Stau1 negatively regulates myogenic differentiation in C2C12 cells. Genes Cells.

[18] Gong C., Kim Y.K., Woeller C.F., Tang Y., Maquat L.E. (2009). SMD and NMD are competitive pathways that contribute to myogenesis: effects on PAX3 and myogenin mRNAs. Genes Dev..

[19] Kretz M. (2013). TINCR, staufen1, and cellular differentiation. RNA Biol..

[20] Cho H., Kim K.M., Han S., Choe J., Park S.G., Choi S.S., Kim Y.K. (2012). Staufen1-mediated mRNA decay functions in adipogenesis. Mol. Cell.

[21] Lebeau G., Maher-Laporte M., Topolnik L., Laurent C.E., Sossin W., Desgroseillers L., Lacaille J.C. (2008). Staufen1 regulation of protein synthesis-dependent long-term potentiation and synaptic function in hippocampal pyramidal cells. Mol. Cell. Biol..

[22] Furic L., Maher-Laporte M., DesGroseillers L. (2008). A genome-wide approach identifies distinct but overlapping subsets of cellular mRNAs associated with Staufen1- and Staufen2-containing ribonucleoprotein complexes. RNA.

[23] Laver J.D., Li X., Ancevicius K., Westwood J.T., Smibert C.A., Morris Q.D., Lipshitz H.D. (2013). Genome-wide analysis of Staufen-associated mRNAs identifies secondary structures that confer target specificity. Nucleic Acids Res..

[24] LeGendre J.B., Campbell Z.T., Kroll-Conner P., Anderson P., Kimble J., Wickens M. (2013). RNA targets and specificity of Staufen, a double-stranded RNA-binding protein in Caenorhabditis elegans. J. Biol. Chem..

[25] Luo M., Duchaine T.F., DesGroseillers L. (2002). Molecular mapping of the determinants involved in human Staufen-ribosome association. Biochem. J..

[26] Tyson J.J., Csikasz-Nagy A., Novak B. (2002). The dynamics of cell cycle regulation. Bioessays.

[27] Tyson J.J., Novak B. (2008). Temporal organization of the cell cycle. Curr. Biol..

[28] Reed S.I. (2003). Ratchets and clocks: the cell cycle, ubiquitylation and protein turnover. Nat. Rev. Mol. Cell Biol..

[29] Nakayama K.I., Nakayama K. (2006). Ubiquitin ligases: cell-cycle control and cancer. Nat. Rev. Cancer.

[30] Pesin J.A., Orr-Weaver T.L. (2008). Regulation of APC/C activators in mitosis and meiosis. *Annu. Rev. Cell Dev. Biol.*.

[31] Glotzer M., Murray A.W., Kirschner M.W. (1991). Cyclin is degraded by the ubiquitin pathway. Nature.

[32] Burton J.L., Tsakraklides V., Solomon M.J. (2005). Assembly of an APC-Cdh1-substrate complex is stimulated by engagement of a destruction box. Mol. Cell.

[33] Pfleger C.M., Kirschner M.W. (2000). The KEN box: an APC recognition signal distinct from the D box targeted by Cdh1. Genes Dev..

[34] Bashir T., Dorrello N.V., Amador V., Guardavaccaro D., Pagano M. (2004). Control of the SCF(Skp2-Cks1) ubiquitin ligase by the APC/C(Cdh1) ubiquitin ligase. *Nature*.

[35] Perroy J., Pontier S., Charest P.G., Aubry M., Bouvier M. (2004). Real-time monitoring of ubiquitination in living cells by BRET. Nat. Methods.

[36] Abrahamyan L.G., Chatel-Chaix L., Ajamian L., Milev M.P., Monette A., Clement J.F., Song R., Lehmann M., DesGroseillers L., Laughrea M. (2010). Novel Staufen1 ribonucleoproteins prevent formation of stress granules but favour encapsidation of HIV-1 genomic RNA. J. Cell Sci..

[37] Harper J.V. (2005). Synchronization of cell populations in G1/S and G2/M phases of the cell cycle. *Methods Mol. Biol.*.

[38] Vassilev L.T. (2006). Cell cycle synchronization at the G2/M phase border by reversible inhibition of CDK1. *Cell Cycle*.

[39] Ferbeyre G., de Stanchina E., Querido E., Baptiste N., Prives C., Lowe S.W. (2000). PML is induced by oncogenic ras and promotes premature senescence. Genes Dev..

[40] Ren S., Rollins B.J. (2004). Cyclin C/cdk3 promotes Rb-dependent G0 exit. *Cell*.

[41] Chatel-Chaix L., Boulay K., Mouland A.J., Desgroseillers L. (2008). The host protein Staufen1 interacts with the Pr55Gag zinc fingers and regulates HIV-1 assembly via its N-terminus. Retrovirology.

[42] Dennis G., Sherman B.T., Hosack D.A., Yang J., Gao W., Lane H.C., Lempicki R.A. (2003). DAVID: database for annotation, visualization, and integrated discovery. Genome Biol..

[43] Sigl R., Wandke C., Rauch V., Kirk J., Hunt T., Geley S. (2009). Loss of the mammalian APC/C activator FZR1 shortens G1 and lengthens S phase but has little effect on exit from mitosis. *J. Cell Sci.*.

[44] Wei W., Ayad N.G., Wan Y., Zhang G.J., Kirschner M.W., Kaelin W.G. (2004). Degradation of the SCF component Skp2 in cell-cycle phase G1 by the anaphase-promoting complex. Nature.

[45] Park H.J., Costa R.H., Lau L.F., Tyner A.L., Raychaudhuri P. (2008). Anaphase-promoting complex/cyclosome-CDH1-mediated proteolysis of the forkhead box M1 transcription factor is critical for regulated entry into S phase. *Mol. Cell. Biol.*.

[46] Almeida A., Bolanos J.P., Moreno S. (2005). Cdh1/Hct1-APC is essential for the survival of postmitotic neurons. *J. Neurosci.*.

[47] Engelbert D., Schnerch D., Baumgarten A., Wasch R. (2008). The ubiquitin ligase APC(Cdh1) is required to maintain genome integrity in primary human cells. Oncogene.

[48] Dephoure N., Zhou C., Villen J., Beausoleil S.A., Bakalarski C.E., Elledge S.J., Gygi S.P. (2008). A quantitative atlas of mitotic phosphorylation. Proc. Natl. Acad. Sci. U.S.A..

[49] Gao D., Inuzuka H., Tseng A., Chin R.Y., Toker A., Wei W. (2009). Phosphorylation by Akt1 promotes cytoplasmic localization of Skp2 and impairs APCCdh1-mediated Skp2 destruction. Nat. Cell Biol..

[50] Rodier G., Coulombe P., Tanguay P.L., Boutonnet C., Meloche S. (2008). Phosphorylation of Skp2 regulated by CDK2 and Cdc14B protects it from degradation by APC(Cdh1) in G1 phase. EMBO J..

[51] Mailand N., Diffley J.F. (2005). CDKs promote DNA replication origin licensing in human cells by protecting Cdc6 from APC/C-dependent proteolysis. *Cell*.

[52] Littlepage L.E., Ruderman J.V. (2002). Identification of a new APC/C recognition domain, the A box, which is required for the Cdh1-dependent destruction of the kinase Aurora-A during mitotic exit. *Genes Dev.*.

[53] Micklem D.R., Adams J., Grunert S., St Johnston D. (2000). Distinct roles of two conserved Staufen domains in oskar mRNA localization and translation. EMBO J..

[54] Martel C., Macchi P., Furic L., Kiebler M.A., Desgroseillers L. (2006). Staufen1 is imported into the nucleolus via a bipartite nuclear localization signal and several modulatory determinants. Biochem. J..

[55] Martel C., Dugre-Brisson S., Boulay K., Breton B., Lapointe G., Armando S., Trepanier V., Duchaine T., Bouvier M., Desgroseillers L. (2010). Multimerization of Staufen1 in live cells. RNA.

[56] Park E., Gleghorn M.L., Maquat L.E. (2013). Staufen2 functions in Staufen1-mediated mRNA decay by binding to itself and its paralog and promoting UPF1 helicase but not ATPase activity. Proc. Natl. Acad. Sci. U.S.A..

[57] Manchado E., Eguren M., Malumbres M. (2010). The anaphase-promoting complex/cyclosome (APC/C): cell-cycle-dependent and -independent functions. *Biochem. Soc. Trans.*.

[58] Hong K.U., Park Y.S., Seong Y.S., Kang D., Bae C.D., Park J. (2007). Functional importance of the anaphase-promoting complex-Cdh1-mediated degradation of TMAP/CKAP2 in regulation of spindle function and cytokinesis. *Mol. Cell. Biol.*.

[59] Lindon C., Pines J. (2004). Ordered proteolysis in anaphase inactivates Plk1 to contribute to proper mitotic exit in human cells. J. Cell Biol..

[60] Seki A., Fang G. (2007). CKAP2 is a spindle-associated protein degraded by APC/C-Cdh1 during mitotic exit. *J. Biol. Chem.*.

[61] Li L., Zhou Y., Sun L., Xing G., Tian C., Sun J., Zhang L., He F. (2007). NuSAP is degraded by APC/C-Cdh1 and its overexpression results in mitotic arrest dependent of its microtubules’ affinity. *Cell. Signal.*.

[62] Wan D., Gong Y., Qin W., Zhang P., Li J., Wei L., Zhou X., Li H., Qiu X., Zhong F. (2004). Large-scale cDNA transfection screening for genes related to cancer development and progression. Proc. Natl. Acad. Sci. U.S.A..

[63] Woodruff J.B., Drubin D.G., Barnes G. (2012). Spindle assembly requires complete disassembly of spindle remnants from the previous cell cycle. Mol. Biol. Cell.

[64] Eliscovich C., Peset I., Vernos I., Mendez R. (2008). Spindle-localized CPE-mediated translation controls meiotic chromosome segregation. Nat. Cell Biol..

[65] Blower M.D., Feric E., Weis K., Heald R. (2007). Genome-wide analysis demonstrates conserved localization of messenger RNAs to mitotic microtubules. J. Cell Biol..

[66] Groisman I., Huang Y.S., Mendez R., Cao Q., Theurkauf W., Richter J.D. (2000). CPEB, maskin, and cyclin B1 mRNA at the mitotic apparatus: implications for local translational control of cell division. Cell.

[67] Raff J.W., Whitfield W.G., Glover D.M. (1990). Two distinct mechanisms localise cyclin B transcripts in syncytial Drosophila embryos. Development.

[68] Lasorella A., Stegmuller J., Guardavaccaro D., Liu G., Carro M.S., Rothschild G., de la Torre-Ubieta L., Pagano M., Bonni A., Iavarone A. (2006). Degradation of Id2 by the anaphase-promoting complex couples cell cycle exit and axonal growth. Nature.

[69] Gabellini D., Colaluca I.N., Vodermaier H.C., Biamonti G., Giacca M., Falaschi A., Riva S., Peverali F.A. (2003). Early mitotic degradation of the homeoprotein HOXC10 is potentially linked to cell cycle progression. EMBO J..

[70] Peters J.M. (2006). The anaphase promoting complex/cyclosome: a machine designed to destroy. *Nat. Rev. Mol. Cell Biol.*.

[71] Christensen K.L., Brennan J.D., Aldridge C.S., Ford H.L. (2007). Cell cycle regulation of the human Six1 homeoprotein is mediated by APC(Cdh1). Oncogene.

[72] Laoukili J., Alvarez-Fernandez M., Stahl M., Medema R.H. (2008). FoxM1 is degraded at mitotic exit in a Cdh1-dependent manner. Cell Cycle.

[73] Marrocco K., Criqui M.C., Zervudacki J., Schott G., Eisler H., Parnet A., Dunoyer P., Genschik P. (2012). APC/C-mediated degradation of dsRNA-binding protein 4 (DRB4) involved in RNA silencing. *PLoS One*.

