# Supplementary material for: Cell cycle-dependent regulation of the RNA-binding protein Staufen1
Source: Nucleic Acids Res. 2014 Jun 7;42(12):7867–83. doi: 10.1093/nar/gku506 (PMC4081104; doi:10.1093/nar/gku506)
Supplement: SUPPLEMENTARY DATA [file supp_42_12_7867__index.html]

Cell cycle-dependent regulation of the RNA-binding protein Staufen1 — Cell cycle-dependent regulation of the RNA-binding protein Staufen1 — SUPPLEMENTARY DATA 

# Cell cycle-dependent regulation of the RNA-binding protein Staufen1

## SUPPLEMENTARY DATA

**Files in this Data Supplement:**

- SUPPLEMENTARY DATA
- SUPPLEMENTARY DATA
- SUPPLEMENTARY DATA
- SUPPLEMENTARY DATA
