# Supplementary material for: Cell cycle-dependent regulation of the RNA-binding protein Staufen1
Source: Nucleic Acids Res. 2014 Jun 7;42(12):7867–83. doi: 10.1093/nar/gku506 (PMC4081104; doi:10.1093/nar/gku506)

Legends

Supplementary figure 1. Quantification of cell cycle markers of figure 1A. As described in the legend of figure 1A, HCT116 cells were grown asynchronously (AS) or were synchronized at the G_1/S transition by a double thymidine block (DTB) and then released by addition of fresh medium. Cell extracts were prepared at different time points post-release as indicated and analyzed by Western blotting (figure 1A). CyclinB, marker of S, G_2 and M phases; AuroraA, marker of G_2 and M phases; MPM2, mitosis protein monoclonal 2 detects a variety of phosphorylated proteins during mitosis. **A)** Relative levels of Stau1 and cell markers at different time points post-release are plotted in the same graph. Data are from figure 1A (Stau1) and supplementary figure 1B (cyclin B1, Aurora A, MPM2). **B)** Quantification of the relative amounts of cell cycle markers at each time point. Standard deviations are shown and statistical analyses (Student's *t*-test) are indicated when significant. The ratios between each protein and β-actin levels were calculated at each time point of 3 independent experiments and the means and standard deviation were plotted relative to that obtained at the highest expression time point, which was arbitrarily set to 1.

Supplementary figure 2. $Stau1^{55}$-$FLAG_3$ expression does not induce cell death or cell cycle exit. HCT116 cells were infected with viruses expressing the empty vector or $Stau1^{55}$-$FLAG_3$ or $Stau1^{\Delta 2}$-$FLAG_3$ as indicated and plated at the same density for 4 days. **(A)** Cell extracts were prepared and analyzed by western blotting. Anti-PARP1 antibody was used to monitor the levels of apoptosis. **(B)** The presence of quiescent cells was quantified by FACS after staining of infected cells with the Hoechst and pyronin Y solutions. Both a representative result and the means and standard deviation of 3 independently performed experiments are provided. **(C)** The percentage of senescent cells was evaluated by microscopy following staining of the cells with β-Gal. The statistical analysis (means and standard deviation) of 3 independently performed experiments is provided.

Supplementary Figure 1

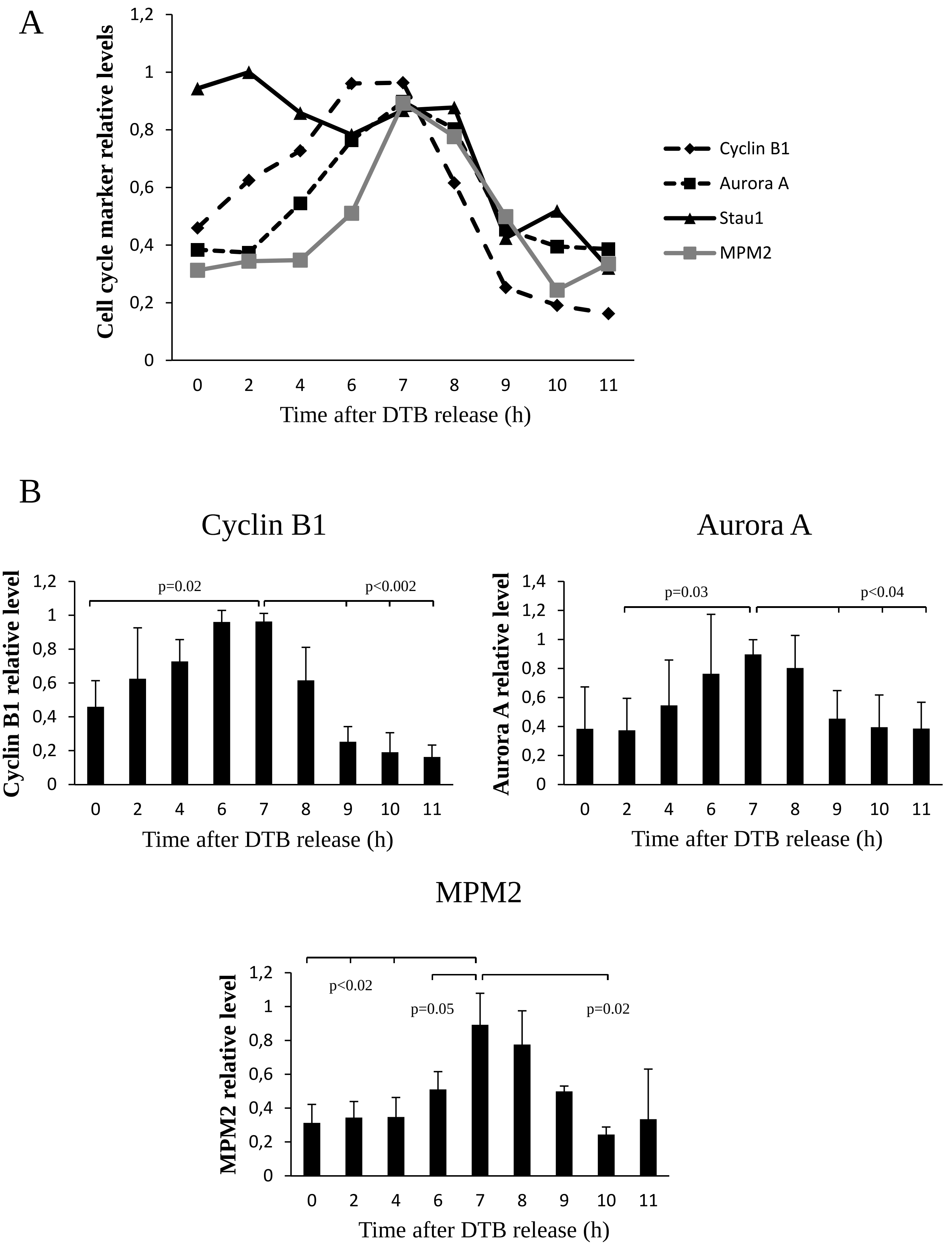

Supplementary Figure 2

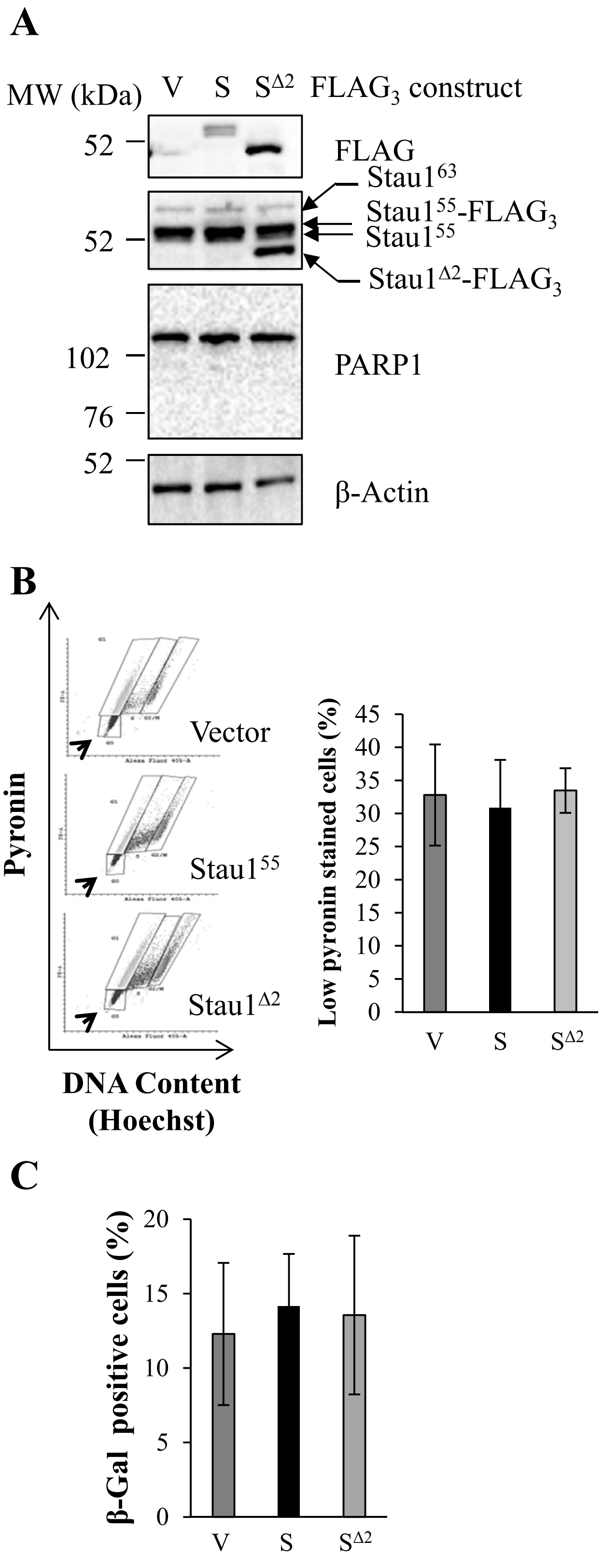

Supplement: SUPPLEMENTARY DATA [file supp_gku506_nar-00783-y-2014-File010.pdf]
